# *Salmonella *induces prominent gene expression in the rat colon

**DOI:** 10.1186/1471-2180-7-84

**Published:** 2007-09-12

**Authors:** Wendy Rodenburg, Jaap Keijer, Evelien Kramer, Susanne Roosing, Carolien Vink, Martijn B Katan, Roelof van der Meer, Ingeborg MJ Bovee-Oudenhoven

**Affiliations:** 1 TI Food and Nutrition, PO Box 557, 6700 AN, Wageningen, The Netherlands; 2 RIKILT Institute of Food Safety, PO Box 230, 6700 AE, Wageningen, The Netherlands; 3 NIZO Food Research, PO Box 20, 6710 BA, Ede, The Netherlands; 4 Vrije Universiteit, Institute of Health Sciences, De Boelelaan 1085, 1081 HV Amsterdam, The Netherlands; 5 Nutrigenomics Consortium, TIFN, PO Box 557, 6700 AN, Wageningen, The Netherlands

## Abstract

**Background:**

*Salmonella enteritidis *is suggested to translocate in the small intestine. *In vivo *it induces gene expression changes in the ileal mucosa and Peyer's patches. Stimulation of *Salmonella *translocation by dietary prebiotics fermented in colon suggests involvement of the colon as well. However, effects of *Salmonella *on colonic gene expression *in vivo *are largely unknown. We aimed to characterize time dependent *Salmonella*-induced changes of colonic mucosal gene expression in rats using whole genome microarrays. For this, rats were orally infected with *Salmonella enteritidis *to mimic a foodborne infection and colonic gene expression was determined at days 1, 3 and 6 post-infection (n = 8 rats per time-point). As fructo-oligosaccharides (FOS) affect colonic physiology, we analyzed colonic mucosal gene expression of FOS-fed versus cellulose-fed rats infected with *Salmonella *in a separate experiment. Colonic mucosal samples were isolated at day 2 post-infection.

**Results:**

*Salmonella *affected transport (e.g. Chloride channel calcium activated 6, H^+^/K^+ ^transporting Atp-ase), antimicrobial defense (e.g. Lipopolysaccharide binding protein, Defensin 5 and phospholipase A2), inflammation (e.g. calprotectin), oxidative stress related genes (e.g. Dual oxidase 2 and Glutathione peroxidase 2) and Proteolysis (e.g. Ubiquitin D and Proteosome subunit beta type 9). Furthermore, *Salmonella *translocation increased serum IFNγ and many interferon-related genes in colonic mucosa. The gene most strongly induced by *Salmonella *infection was Pancreatitis Associated Protein (*Pap*), showing >100-fold induction at day 6 after oral infection. Results were confirmed by Q-PCR in individual rats. Stimulation of *Salmonella *translocation by dietary FOS was accompanied by enhancement of the *Salmonella*-induced mucosal processes, not by induction of other processes.

**Conclusion:**

We conclude that the colon is a target tissue for *Salmonella*, considering the abundant changes in mucosal gene expression.

## Background

Foodborne infections cause a major burden on public health services and represent significant costs in many countries. *Salmonella *infection is one of the most common and widely distributed foodborne diseases and can be severe in the young, the elderly and patients with weakened immunity. *Salmonella enteritidis *is the most frequently isolated serotype, causing gastroenteritis in most humans and systemic infection in a subpopulation [1,2]. The precise mechanisms of *Salmonella-*host interaction *in vivo *at early time points after infection are not well known. Insight in pathogen-induced host processes *in vivo *could help to design therapeutic or nutritional strategies for infection prevention. An approach to investigate the effects of a pathogen on host target cells is the use of microarrays that contain the whole genome of the host. This broad approach can reveal biological processes affected by the pathogen. The rat is a good model to study *Salmonella enteritidis*-induced host processes, since salmonellosis in the rat shares many features of human disease [3]. Besides gastroenteritis, a self-limiting systemic infection is observed in rats. The ileum is thought to be the main site of *Salmonella *invasion in both humans and rats [4]. For this reason we have previously studied *Salmonella*-induced gene expression in the ileum of rats. This study showed that *Salmonella *affects only a small number of genes at early time points post-infection [5]. Carbohydrate transport, antimicrobial defense and detoxification were the main affected biological processes. At later time points large numbers of inflammation genes were found to be up-regulated in the ileal mucosa. The colon mucosa is supposed to be protected from *Salmonella *colonization by the abundant intestinal microflora. Pathogens entering the colon have to compete for nutrients and binding places with the endogenous flora. However, biopsies taken from humans during an infection with nontyphoid *Salmonella *setorypes suggest that the colon is involved in *Salmonella *infections [6-8]. As most studies focus on the ileum, which is thought to be the most likely site of translocation, only little information is available on *Salmonella *translocation in the large intestine [9]. Besides indications from studies on biopsies, we have another reason to suspect colonic involvement in *Salmonella *infection pathology. We have shown earlier that diets supplemented with prebiotics such as fructo-oligosaccharides (FOS), lactulose and inulin consistently increased intestinal *Salmonella *translocation in rats [10-13]. As fermentation of FOS, and other prebiotics, occurs in cecum and colon and is very limited in the ileum of humans [14] and rats [15], it is unlikely that prebiotics facilitated translocation of *Salmonella *at that particular site. This is supported by the absence of ileal inflammation in FOS-fed and *Salmonella*-infected rats in contrast to profound cecal and colonic inflammation [11]. To extend the current limited evidence indicating colonic involvement in *Salmonella *infection, we used transcriptional profiling to investigate genes and biological processes in the rat colonic mucosa affected by *Salmonella*. We first studied colonic mucosal gene expression responses at days 1, 3 and 6 after oral *Salmonella *infection of rats using whole genome microarrays and Q-PCR. In a second infection experiment, we studied whether the increased translocation of *Salmonella *by dietary FOS was reflected by amplification of *Salmonella-*induced gene expression changes in the colon.

## Results

### Time course infection study

#### General infection characteristics

**Figure 1:**
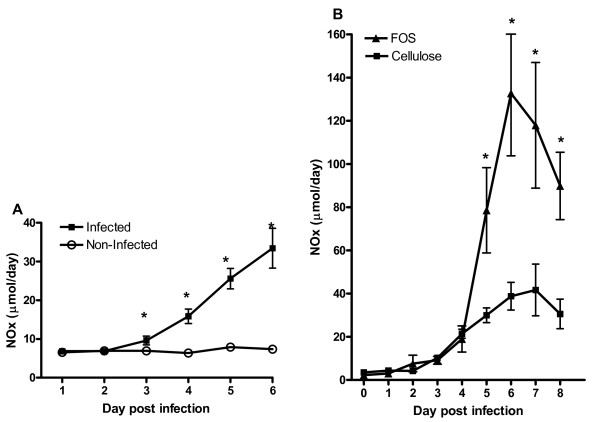
Sum of urinary nitrate and nitrite (NO_x_) excretion in the non-infected (○), infected (■) groups of the time course infection study (A). And the urinary NO_x _excretion in the cellulose infected (■) and in the fructo-oligosaccharide (FOS) infected (▲) groups in the dietary infection study (B). Infected rats were orally challenged with *S. enteritidis *on day 0. Results are expressed as mean ± SEM (n = 8 in the time course infection study and n = 6 in the dietary infection study). * p < 0.05.

In agreement with previous studies, food consumption and growth of the Wistar rats were not affected by *Salmonella *infection [16]. *Salmonella *translocation to mesenteric lymph nodes was observed at days 1, 3 and 6 (table 1). This implies that at day 1, *Salmonella *has already crossed the intestinal barrier. In agreement with previous studies [16,17], *Salmonella *was detected in the spleen at days 3 and 6 and in the liver at day 6 (table 1). Urinary NO_x _excretion, a parameter of systemic infection, was found to be increased from day 3 onwards (figure 1a).

**Table 1:** Viable *Salmonella *counts in feces, mesenteric lymph nodes, spleen, and liver of rats 1, 3 and 6 days post infection

		*Salmonella *(logCFU/g)^a^		
		
	Control	Day 1 p.i.	Day 3 p.i.	Day 6 p.i.
Feces	N.D.	7.22 ± 0.19	5.92 ± 0.24	6.04 ± 0.32
MLN	N.D.	3.38 ± 0.43	5.85 ± 0.13	5.44 ± 0.05
Spleen	N.D.	N.D	3.20 ± 0.33	3.49 ± 0.05
Liver	N.D.	N.D	N.D	2.45 ± 0.12

^a ^The rats were orally infected with 3 × 10^9^colony-forming units of *S. enteritidis *or control treated. *Salmonella *counts are expressed in log values as means ± SEM (n = 8). N.D. = not detected.

#### Salmonella-induced processes in colon mucosa

**Figure 2:**
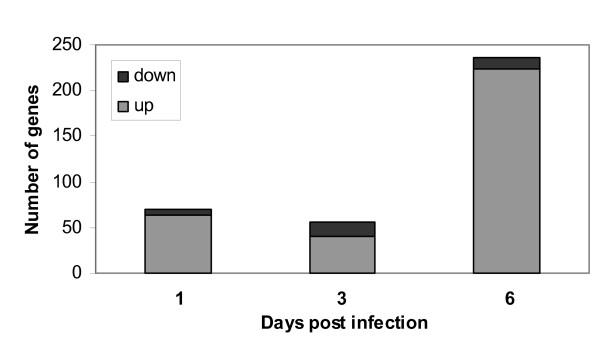
The number of differentially expressed genes with a fold change greater than 2 in colon mucosa of rats at days 1, 3 or 6 after oral infection with *Salmonella *or control treatment.

To identify *Salmonella*-regulated processes, microarray-based gene expression profiling of colonic mucosa at days 1, 3 and 6 days p.i. was performed. The arrays contained 44000 spots of which 32783 spots exceeded >2 times the background value and were included in the analysis. *Salmonella *changed the expression of 330 genes >2-fold at least at one of the three time points studied. At days 1 and 3 p.i. comparable numbers of genes (70 and 57 genes, respectively) were affected by *Salmonella *infection in comparison with non-infected rats, while at day 6 approximately four times more genes were affected (figure 2). This corresponded with progression of the infection as observed by the organ cultures and urinary NO_x_ excretion as mentioned above. At all time points studied, most genes showed increased expression upon *Salmonella *infection, whereas only a small percentage of total regulated genes were down-regulated (10% at day 1, 27% at day 3, 5% at day 6 figure 2). The genes that changed more than 2-fold at any time point (FC > 2 infected/non-infected) were classified into biological processes according to gene ontology terminology [18,19]. Not all genes are annotated to GO processes. Forty percent of the genes on the array were annotated to GO processes. Therefore we manually supplemented the significant processes (p < 0.001) with the remaining significant genes using biological databases and scientific literature. To prevent the occurrence of false positive genes, and over-interpretation of biological processes affected by *Salmonella*, we focused on biological processes with at least three genes exceeding the cut-off FC > 2.0. Additionally, we observed that the genes within one biological process showed comparable patterns of expression (table 2), which strongly indicates that these processes are truly affected by *Salmonella*.

**Table 2:** Processes regulated in colon by *Salmonella *at days 1, 3 and 6 after oral *Salmonella *infection

Gene Name	Gene symbol	Sequence ID	Fold Change infected vs non-infected rats on different days p.i.			
			
			Time course infection study^a^			Dietary infection study^b^
			
			Day 1	Day 3	Day 6	Day 2
**Transport**						
*Ion transport*						
Chloride channel calcium activated 6	*Clca6*	NM_201419	**2,3**	**2,2**	**3,7**	*2,3*
Calcium channel, voltage-dependent, alpha 1I subunit	*Cacna1i*	NM_020084	**2,2**	1,5	**-**	*1,2*
Solute carrier family 4, member 1 (Slc4a1), anion exchanger	*Slc4a1*	NM_012651	**2,0**	1,7	**-**	*1,0*
Atp-ase, H+/K+ transporting, nongastric, alpha polypeptide	*Atp12a*	NM_133517	**2,8**	**-**	**2,0**	*1,1*
Solute carrier family 20 (phosphate transporter), member 1	*Slc20a1*	NM_031148	**2,1**	**-**	**-**	*-1,2*
Solute carrier family 15 (oligopeptide transporter), member 1	*Slc15a1*	NM_057121	**2,0**	**1,5**	**1,6**	*0,9*
Transporter 1, ATP-binding cassette, sub-family B	*Mdr/Tap1*	NM_032055	**-**	1,8	**2,8**	*1,4*

**Oxidative stress**						
Dual oxidase 2	*Duox2*	NM_024141	1,9	**2,4**	**2,8**	*1,6*
Glutathione peroxidase 2	*Gpx2*	NM_183403	-	**2,3**	**3,0**	*2,2*
Xanthine dehydrogenase	*Xdh*	NM_017154	-	1,8	**2,5**	*1,4*

**Immune response**						
Rat class III Fc-gamma receptor	*Fcgr3*	M64368	**2,1**	1,6	-	*ND*
Immunoglobulin superfamily, member 4	*Igsf4d*	XM_340958	**2,1**	1,6	1,4	*1,1*
Rat MHC class I truncated cell surface antigen	*RT1-Aw2*	M10094	**2,0**	-	1,9	*1,0*
Interleukin enhancer-binding factor 1	*Ilf1*	XM_221212	**2,0**	1,5	-	*1,1*
Colony stimulating factor 2 (granulocyte-macrophage)	*Csf2*	XM_340799	**2,0**	1,6	-	*1,2*
Interleukin 1 alpha	*Il1a*	NM_017019	1,8	**2,0**	**2,3**	*1,4*
Interleukin 1 beta	*Il1b*	NM_031512	**-**	**2,1**	**4,0**	*2,6*
TRAF2 binding protein	*T2bp*	NM_001014044	1,8	**2,8**	**4,4**	*2,4*
Toll-like receptor 2	*Tlr2*	NM_198769	-	1,5	**2,3**	*1,3*
***Antimicrobial defense***						
Lipopolysaccharide binding protein	*Lbp*	NM_017208	1,9	1,8	**2,2**	*1,3*
Defensin 5 precursor (Enteric defensin)	*RD-5*	XM_214386	-1,9	-1,6	-1,6	*ND*
Phospholipase A2, group IIA (platelets, synovial fluid)	*Pla2g2a*	NM_031598	**3,4**	**5,2**	**10,5**	*7,3*
***Inflammatory response***						
Pancreatitis-associated protein	*Pap*	NM_053289	**11,4**	**44,6**	**114,2**	*17,7*
Tissue-type transglutaminase	*Tgm2*	NM_019386	-	**2,3**	**4,9**	*1,8*
Regenerating islet-derived 3 gamma	*Reg3g*	NM_173097	-	**2,3**	**4,3**	*1,9*
Nitric oxide synthase 2, inducible	*Nos2*	NM_012611	-	1,6	**4,0**	*ND*
S100 calcium binding protein A8 (calgranulin A)	*S100a8*	NM_053822	-	1,8	**2,4**	*1,4*
Calprotectin ⟨						
S100 calcium binding protein A9 (calgranulin B)	*S100a9*	NM_053587	-	1,7	1,9	*1,3*

**Interferon**						
Interferon-induced guanylate-binding protein 1	*Gbp1*	XM_221883	**2,4**	1,9	**2,2**	*1,3*
Interferon gamma inducible protein	*Ifi47*	NM_172019	1,7	**2,7**	**7,3**	*2,6*
Guanylate binding protein 2, interferon-inducible	*Gbp2*	NM_133624	1,6	**2,4**	**3,1**	*1,7*
Interferon-induced protein	*Ifit2*	NM_001024753	1,5	1,7	**3,4**	*1,3*
Interferon-stimulated protein	*G1P2*	XM_216605	-	1,6	**4,1**	*1,5*
Immunity-related GTPase family, M	*Irgm*	NM_001012007	-	1,8	**3,7**	*1,4*
Signal transducer and activator of transcription 1	*Stat1*	NM_032612	-	1,7	**3,6**	*1,8*
Interferon regulatory factor 7	*Irf7*	XM_215121	-	1,5	**2,6**	*ND*
Alpha-interferon	*Ifna*	XM_233145	**-**	**2,0**	**-**	*ND*

**Proteolysis**						
Ubiquitin D	*Ubd*	NM_053299	1,7	**2,5**	**15,2**	*3,4*
Proteosome (prosome, macropain) subunit, beta type 9	*Psmb9*	NM_012708	-	**2,0**	**3,7**	*2,0*
Protease, serine, 22	*Prss22*	XM_220222	-	**2,0**	**2,6**	*1,6*
Potential ubiquitin ligase	*Herc6*	XM_342700	-	1,7	**3,3**	*1,5*
Proteasome (prosome, macropain) subunit, beta type 10	*Psmb10*	XM_214687	-	1,5	**2,1**	*1,5*

^a^Values in bold exceed cut-off value FC > 2 or FC < -2. Values -1,5 < FC < 1,5 are indicated by (-).

^b ^Fold Change infected vs non-infected rats fed a cellulose diet at day 2 p.i (obtained from the dietary infection study). All fold changes are shown. Genes not detected in this independent study are indicated by ND.

**Figure 3:**
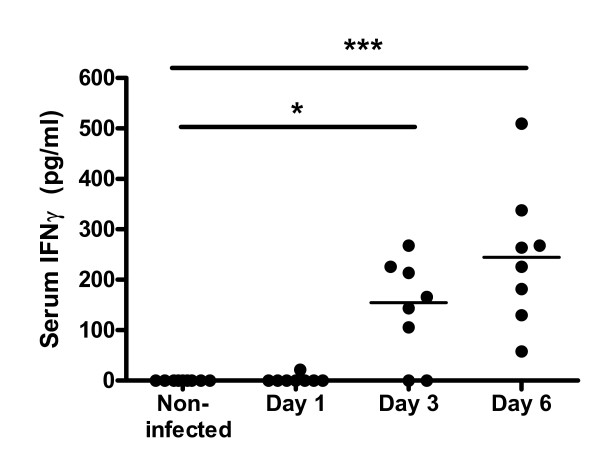
Serum IFNγ levels before and after infection (days 1, 3 and 6 p.i.). Each dot represents an individual rat. Group medians are presented by a black line. * p < 0.05, ***p < 0.001.

We focused on the early *Salmonella*-induced gene expression changes occurring at days 1 and 3 p.i. Presumably, these early modulated genes are more related to *Salmonella*-induced primary changes than gene expression at day 6 which is a secondary result of *Salmonella*-induced inflammation. Genes affected >2-fold on day 1 and/or day 3 p.i. that could be related to a biological process are shown in table 2. The biological processes that contained 3 or more modulated genes were transport, oxidative stress, immune response, antimicrobial defense, inflammatory response, interferon pathways and proteolysis. For more insight into these processes, genes that changed >2-fold at day 6 p.i. and also showing a >1.5-fold induction at day 1 or 3 p.i. were also added to this table. Genes that changed >2-fold on day 6 p.i. only are shown in Additional File 1. The gene most affected by *Salmonella *infection in the colon was pancreatitis associated protein (*Pap*), showing 11, 45 and 114 fold induction at days 1, 3 and 6 respectively. Seventy genes changed >2-fold at day 1 p.i., of these genes 7 encoded for transporters and 5 genes encoded for immune response proteins (table 2). At day 3 p.i., 57 genes showed FC > 2, again including genes encoding for immune response proteins. Induced expression of Interleukin 1β and 1α indicates activation of an inflammatory response. Induction of dual oxidase 2 and glutathione peroxidase 2 suggest oxidative stress in the colonic mucosa. At day 6 more than 200 genes were induced more than 2-fold in infected mucosa compared with non-infected mucosa (Additional File 1). Most of these genes were related to immune and inflammatory responses. Processes related to inflammation-induced damage and repair, such as connective tissue remodeling and chemo-attraction also showed clear induction at day 6 p.i. To exclude the possibility that the observed changes were due to cellular changes of the mucosa, we analyzed expression differences of cell-type specific genes [20,21] (Additional File 2). As transporters are most likely expressed by enterocytes [22], we examined expression of enterocyte specific genes (*Fabp2, Vil2, Alpi2*). These genes showed diverse regulation, indicating that the increased expression of transporters at day 1 is not due to altered enterocyte composition in the mucosal samples. A similar observation was found for Goblet cell specific genes (*Muc2, Muc3, Tff1, Tff3*) and Paneth cell specific genes (*RD-5 *and *Pla2g2a*). Expression of leukocyte specific genes was not altered at early timepoint, a mild increase was observed at day 6 p.i. Together this indicated that the observed *Salmonella *induced gene expression changes did not result from changes in cellular composition of the mucosa. This is in agreement with histology results from earlier *Salmonella *infection experiments, showing no or only minor deviations in intestinal mucosal architecture from healthy control slides (data not shown). This is further supported by the relatively constant expression of a group of well known housekeeping genes (Additional File 2). The largest group of related genes induced by *Salmonella *infection in colon mucosa is related to interferon pathways as more than 20 IFNγ-regulated genes showed increased expression at at least one time point studied. The IFNγ-induced gene expression was most prominent at day 6 p.i, but already from day 1 onwards induction of several IFNγ-inducible GTPases (*Gbp1, Gbp2, Ifi47, Ifit2*) was seen (table 2). Furthermore at day 3 p.i. (table 2) induction of two members of the IFNγ-signaling pathway (*Stat1 *and *Irf7*) was observed. Despite induction of many interferon-related genes, increased expression of IFNγ mRNA itself could not be detected (changed 1.1-fold at days 1 and 3, and 1.3-fold at day 6 p.i.). IFNγ protein concentrations were measured in individual serum samples. IFNγ was not detected in serum of non-infected rats (all timepoints) and at the first day after *Salmonella *administration to rats. However, from day 3 p.i. serum IFNγ increased (figure 3). The serum IFNγ most probably originated from peripheral immune activation, as the increase in serum IFNγ followed the same trend as the increase in *Salmonella *CFU's in peripheral organs (table 1). In the time course infection study, the kinetics of urinary NO_x_ excretion are reflected by *Nos2 *gene expression in colonic mucosal with a small 1.6-fold induction at day 3 p.i. and a 4 fold induction at day 6 p.i. (table 2).

#### Q-PCR confirmation of Salmonella-induced gene-expression

**Figure 4:**
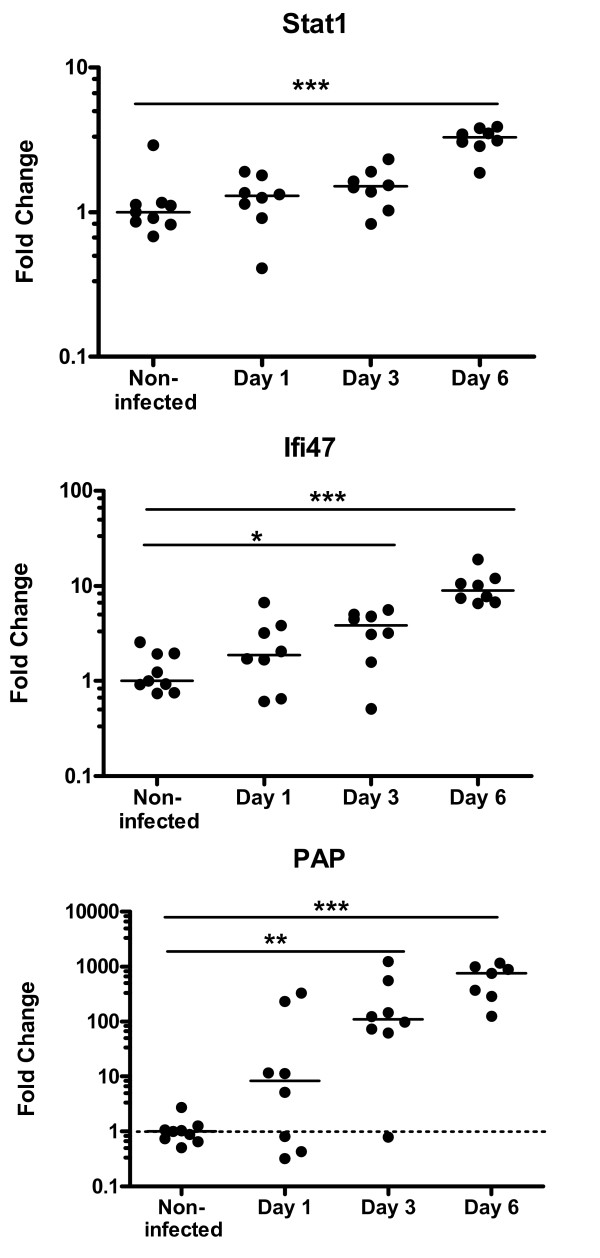
Individual expression of two interferon-related genes and *Pap *in the colon mucosa at different time points after *Salmonella *infection or control treatment. Genes expression is quantified by Q-PCR, using *Rps26 *as reference gene (using *Arf1 *as reference gene showed similar results; data not shown). Each dot represents an individual rat. Dotted line indicate lowest mRNA standard. Medians are presented by a black line. The median value of the uninfected group was set at 1.0. Y-axis is at log_10 _scale. * p < 0.05, **p < 0.01, ***p < 0.001.

To determine inter-individual variation in gene expression within treatment groups, RNA from the colon of individual animals was analyzed by Q-PCR. We chose individual confirmation of *Stat1 *and *Ifi47 *to gain insight in inter individual interferon response as we also focused on the individual protein levels of IFNγ. Confirmation of PAP was chosen to obtain insight in the individual kinetics of the most strongly induced gene in colon mucosa at all time points. Q-PCR analysis showed rather large inter-individual variation among the outbred rats. *Pap *expression levels in the non-infected colonic mucosa were near detection level, which made it difficult to determine precise fold changes. Nevertheless, the Q-PCR analysis of the three genes examined clearly confirmed the gene expression changes observed in the microarray analysis (figure 4). To further validate the array data of the time course infection study we compared the gene expression changes of day 1 and 3 p.i. with gene expression data obtained from the independent dietary infection study at day 2 p.i. (table 2). Similar biological processes were induced at early timepoints in both studies. At individual gene expression level several transporter genes (*Cacna1i, Slc4a1, Slc15a1*) and immune response genes (*Igsf4d, Ilf1, Csf2*) showed no overlap possibly due to infection kinetics. However gene expression results of day 3 and day 2 p.i. largely overlapped (table 2).

### Dietary infection study

#### General infection characteristics

Food consumption and growth of the Wistar rats on both cellulose and FOS diet were similar, before and after infection. The section was performed on day 2 p.i. because similar effects on gene expression at days 1 and 3 were observed in the time course infection study. Furthermore we were interested in the primary responses as we expect that diet will mainly influence early events such as attachment to the mucosa and translocation of the pathogen. These events occur for the most part luminal where direct interaction between dietary components, pathogens and mucosa take place, while later phases merely reflect secondary infection and inflammation responses. At day 3 p.i. the serum IFNγ concentration and the amount of translocated *Salmonella *in the spleen are already high, which indicates systemic infection. At day 1 p.i. no serum IFNγ or *Salmonella *translocation to the spleen was observed. We chose day 2 p.i. as this seems the appropriate time point to study the effects of FOS on early *Salmonella*-induced changes. *Salmonella *colonization was quantified by determination of colony-forming units (CFU/g) in fresh fecal samples with time. At day 1 *Salmonella *levels were not significantly different between cellulose and FOS-fed animals (7.23 ± 0.11 and 7.10 ± 0.22, respectively). At day 2 FOS-fed rats had more *Salmonella *in feces than their cellulose counterparts (7.25 ± 0.25 and 6.53 ± 0.25, respectively; p < 0.05). *Salmonella *translocation to mesenteric lymph nodes and spleen was not significantly different in the FOS group compared to the cellulose group when quantified by CFU. Viable *Salmonella *counts in MLN were 5.96 ± 0.08 in the cellulose group and 6.19 ± 0.10 log_10 _CFU/g in the FOS group. Numbers in spleen were 2.85 ± 0.14 (cellulose) and 2.98 ± 0.16 log_10 _CFU/g (FOS). Counts in liver were under the detection limit of 10^2 ^CFU/g tissue in the cellulose and FOS group. These numbers are comparable to those observed in the time course infection study at day 3 and highly similar to numbers observed in earlier studies which showed increased translocation in FOS-fed rats at later time points after infection [23]. To observe long term effects of FOS on *Salmonella *translocation in this study, urinary NO_x_ excretion with time was determined in additional groups of rats. Urinary NO_x_ excretion of FOS-fed rats increased to 132 μmol/d at day 6 p.i. and started to decline towards baseline levels thereafter (figure 1B). Peak urinary NO_x_ excretion of infected rats fed the cellulose diet was just one third of the level reached by the infected rats fed the FOS diet, i.e. 41 μmol/day (figure 1B). The NO_x _values for the cellulose diet are similar to those obtained in the time course infection study (figure 1A). The kinetics of urinary NOx excretion were similar in both diet groups, but total infection-induced urinary NO_x_ excretion was higher in the FOS group indicating enhanced *Salmonella *translocation.

#### Effect of dietary FOS on Salmonella-induced mucosal genes in colon mucosa

The rats of the time course infection study and the rats in the dietary infection study on cellulose diet showed a comparable urinary NO_x _excretion and thus *Salmonella *translocation response. Despite the fact that the two studies were separately performed and different time points were studied, the identified biological processes affected by *Salmonella *at day two p.i. were comparable to processes observed at days 1 and 3 p.i. Furthermore both studies showed that more genes were up-regulated than down-regulated by *Salmonella*. For detailed analysis, we focused on the most robust genes, i.e. genes that showed similar *Salmonella *induced regulation in the two independent studies. We choose a threshold of FC < 1.5 for both studies, which is less stringent than the threshold we choose for analysis within one study (FC < 2.0). We feel that this is legitimate, as genes with small but similar regulation in two completely independent studies are less likely to be selected by chance. Thirty-one genes fulfilled this criterion, 26 were up-regulated and 5 down-regulated. Eighteen of the up-regulated genes were categorized to the same processes found to be modulated by *Salmonella *in the colonic mucosa in the time course infection study, i.e. the transporter *Clca6*, the oxidative stress genes *Gpx2 *and *Duox2*, the immune response genes *Il1b and T2bp*, the antimicrobial defense gene *Pla2g2a *the inflammatory response genes *Pap, Tgm2 and Reg3g*, the interferon related genes *Ifi47, Gbp2, Iigp2, P47Iigp, Stat1, G1p2 *and the proteasome related genes *Psmb9, Prss22, Psmb10, Ubd *(Table 2). The other 8 up-regulated genes which could not be grouped to a specific process were Palmitoyl-protein thioesterase, Schlafen 3 (*Slfn3*), Tripartite motif protein 15 (*Trim15*), Aquaporin 3 (*Aqp3*) and four unknown genes. The 5 down-regulated genes were Heat shock protein 70 kD 1A (*Hspa1a*), Resistin like alpha (*Retnla*), Resistin like gamma *(Retnlg*), Collectin sub-family member 10 (*Colec 10*) and Mammalian suppressor of Sec4 (*Mss4*). Not all processes that were identified in the time course infection study at both days 1 and 3 p.i. were confirmed in the dietary infection study at day 2 p.i. (table 2). This was the case for two processes, namely transport (*Cacna1i, Slc4a1, Atp12a, Slc15a1*) and immune response (*Igsf4d, RT1, Ilf1, Csf2, Il1a*). Furthermore the antimicrobial defense gene *Lbp *and two interferon pathway genes (*Gbp1, Ifit2*) were not confirmed.

**Figure 5:**
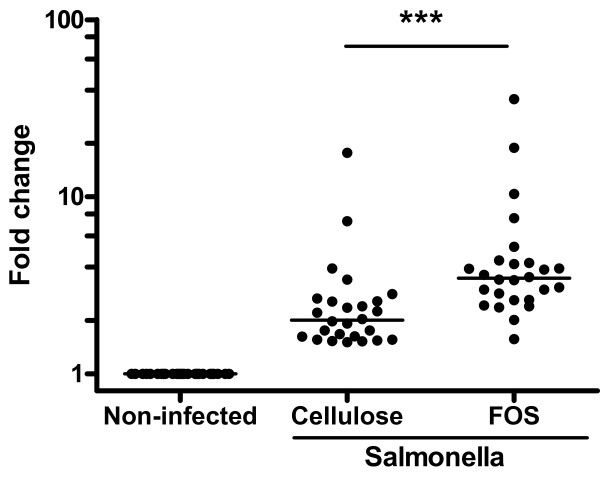
Expression level of most consistent *Salmonella*-target genes in colon mucosa of rats fed a cellulose diet or a FOS diet. The gene expression is obtained from micro array analysis of pooled colonic mucosa samples collected at day 2 post-infection. Each dot represents a gene. The median value of each gene in the uninfected group is set to 1.0. Y-axis is at log_2_scale. ***p < 0.001.

To examine whether our choice for FC > 1.5 was legitimate, we studied whether application of threshold FC > 1.3 and FC > 1.7 resulted in identification of the same processes as identified with FC > 1.5. The general picture of processes affected was the same for FC > 1.5 and FC > 1.7. However, with FC > 1.3 more genes could be included in processes identified with FC > 1.5, such as the interferon response and proteolysis (data not shown). However, many other genes could not be grouped into (new) specific biological processes, indicating that a cut-off FC > 1.3 might be too flexible and results in introduction of false positive processes, probably not related to the treatment. Therefore, we choose FC > 1.5 for further analysis. To investigate the effects of FOS on *Salmonella *infection in the colon, we studied the expression of *Salmonella-*induced colonic mucosal genes in infected rats fed the cellulose diet versus infected rats fed the FOS supplemented diet. The five genes that were consistently downregulated by *Salmonella *in both studies (*Hspa1a, Retnla, Retnlg, Colec 10 and Mss4*) were not further influenced by FOS (equal gene expression in cellulose- and FOS-fed infected rats). For initiating early mucosal events after *Salmonella *infection (e.g. chemo attraction of inflammatory cells) increases in epithelial gene expression may be more important than decreases[24,25]. We focused on the 26 genes which showed a consistent increase in gene expression after *Salmonella *infection of FC > 1.5 in both studies. All 26 genes consistently induced by *Salmonella *infection in the colon mucosa showed a further up-regulation in colon mucosa of *Salmonella *infected rats fed FOS (figure 5). The effect of FOS on the cluster of *Salmonella *affected genes was statistically significant. To asses the inter-individual gene expression in the dietary infection study we selected genes from several *Salmonella *modulated process for individual Q-PCR confirmation: *Clca6, Gpx2, Il1b, Pla2g2a, Pap, Tgm2, Stat1, Gbp2 *and *Ifi47*. Q-PCR of the selected genes in individual samples showed high inter-individual variation but confirmed the fold changes of the microarray study using pooled samples (table 3). The confirmed *Salmonella *induced gene expression changes were significant (p < 0.05) for 7 of the 9 genes, except for Tgm2 (p = 0.09) and Stat1 (p = 0.08). Examination of FOS-fed versus cellulose-fed infected groups on individual gene level showed a significant increase of *Clca6 *and *Pla2g2a*. Expression of *Gpx2, Il1*β and *Tgm2 *was >1.5-fold increased by FOS feeding in comparison to cellulose feeding but this was not statistically significant. The genes *Pap, Stat1, Ifi47 *and *Gbp2 *showed non-significant and small increases of 1.1–1.4 fold. In t-testing each gene is tested independently, the FOS vs cellulose effect was not statistically significant for each independent gene. However, FOS significantly increased expression of the cluster of the 26 Salmonella induced genes (see figure 5 and Additional File 3). We also looked at overall gene expression differences between cellulose- and FOS-fed rats at day 2 after *Salmonella *infection (Additional File 3). This was done to determine whether the stimulated translocation in FOS-fed rats resulted in additionally affected genes or biological processes not induced by *Salmonella *in the cellulose groups. Twenty genes were induced by *Salmonella *>2-fold in cellulose-fed infected rats. In the FOS-fed infected rats 72 genes were induced by *Salmonella *>2-fold. Seventeen genes overlapped between these two diet groups. Detailed analysis of the genes exclusively induced (>2-fold) in the FOS-fed group showed that those could be categorized in the same processes identified earlier (table 2 and Additional File 3). Obviously, the induced translocation of *Salmonella *by FOS supplementation did not affect other processes than those already identified in *Salmonella *infected rats on a cellulose diet. However, more genes of the same processes and higher fold-changes were noticed in the colonic mucosa of infected rats on the FOS diet.

**Table 3:** Q-PCR analysis of *Salmonella *-induced colonic mucosal gene expression of rats on a cellulose or a FOS diet (day 2 p.i.)

	Relative gene expression ^a^		
	
	Non-infected ^b^	Infected	
		
Gene symbol		Cellulose	FOS
*Clca6*	1 (0.9–1.3)	1.6 (1.4–2.2)	3.7 (2.8–4.1)
*Pla2g2a*	1 (0.8–1.3)	4.6 (2.3–7.1)	8.8 (6.1–21.1)
*Gpx2*	1 (0.8–1.2)	2.1 (1.7–2.4)	3.2 (1.3–4.8)
*Il1b*	1 (0.6–1.1)	2.0 (1.3–3.4)	3.8 (0.6–5.4)
*Tgm2*	1 (0.5–1.2)	1.6 (0.7–4.4)	10.2 (0.8–20.6)
*Pap*	1 (0.4–2.4)	236 (68–326)	288 (13–1162)
*Stat1*	1 (0.7–1.2)	1.2 (1.1–2.2)	1.5 (0.7–4.0)
*Ifi47*	1 (0.8–1.2)	3.9 (2.1–6.4)	5.6 (1.6–18.4)
*Gbp2*	1 (0.8–1.4)	2.0 (1.1–3.2)	2.1 (0.5–9.7)

^a^The expression of genes is analyzed by QPCR, using *Rps26 *as reference gene (using Arf1 as reference gene gave similar results; data not shown). Data are represented as median (25% percentile–75% percentile).

^b^The median value of the non-infected group is set to 1.0.

*Nos2 *gene expression in colonic mucosa was below detection levels in the dietary infection study. As significant differences in NOx excretion between infected cellulose- and FOS-fed rats were observed from day 5, no differences at *Nos2 *gene expression were expected at day 2 p.i. Serum IFNγ was not detected at day 2 p.i., neither in infected cellulose-fed rats nor in infected FOS-fed rats.

## Discussion

### Colon is an infection target

This study shows quick and profound gene expression changes in the rat colon mucosa upon oral *S. enteritidis *infection, which implicates that not only the ileum, but also the colon, is a target for *Salmonella *infection. The earliest responses were noticed on mucosal transport and antimicrobial defense. The most responsive gene is *Pap*, which showed an 11-fold induction in colon mucosa on the first day after infection and increased to over 100 fold at day 6. At later timepoints, the most notable process affected is interferon-related. Colonic genes consistently induced by *Salmonella *infection in two independent studies, were all further enhanced by FOS supplementation, a known stimulus of colonic bacterial fermentation. *Salmonella*, ingested with contaminated foods or drinks, is thought to colonize the distal small intestine and to translocate through ileal Peyer's patches to extra-intestinal organs [4,26]. Several observations suggest that other parts of the intestine are also involved in *Salmonella *infection. High numbers of *Salmonella *are found in the cecum and colon of orally infected rats [4,26] as well as pigs [27]. In humans *Salmonella *commonly affects the small intestine, but colonic involvement of *S. enteritidis *has been reported in humans [6,7,28] and may play an important role in induction of diarrhea [6]. Studies describing mucosal invasion via the paracellular and transcellular route [29,30] also suggest that translocation of *Salmonella *species to the systemic circulation is not restricted to the ileal Peyer's patches. Our studies on the effects of prebiotics on resistance of the host to *Salmonella *infection also point to the colon as invasion site [11]. Together results from literature and those presented here indicate that the colon is one of the targets for *Salmonella *infection.

### Interferon-gamma response

The increase of many IFNγ-regulated genes in the *Salmonella-*infected colon in the present *in vivo *study actually confirms the earlier suggested role of IFNγ in relation to host defense against *Salmonella*. Serum IFNγ levels increase in mice infected with *Salmonella *by oral or intraperitoneal route [31-34]. IFNγ is produced by natural killer cells, CD4 Th1 cells and CD8 cytotoxic lymphocytes [35-37]. IFNγ most likely exerts its function in host defense by activation of macrophages which can kill *Salmonella *[38]. In our *Salmonella *time course infection study, more than 20 IFNγ-related genes were up-regulated (table 2). This did not coincide with an increased IFNγ mRNA level at any of the time points studied. In addition, we could not detect IFNγ protein in *Salmonella*-infected colons (data not shown). Serum levels of this pro-inflammatory cytokine were undetectable at day 1 p.i. but rose steadily from day 3 p.i. with large inter-individual variation in the magnitude of response (figure 3). Despite the lack of detectable IFNγ protein in colonic mucosa and in serum at day 1, we did observe increased expression of genes in the INFγ induced pathway at that timepoint. These genes are most likely activated by INFγ [39]. We can not fully exclude that dilution of IFNγ-producing cells in the heterogeneous cell population of mucosal scrapings has lead to undetectable levels of this regulatory cytokine in the present study. At the later timepoints, serum IFNγ is strongly increased, whereas mucosal IFNγ remained below detection levels at all timepoints. This may suggest that systemic rather than colonic IFNγ seems to be the trigger for the later activation of IFNγ-related genes and -processes in colonic mucosa upon *Salmonella *infection. However, dilution of IFNγ producing cells in colonic tissue to undetectable levels could also account for this later time point.

As many as 1200 genes are known to be regulated by IFNγ. Their gene products are mediators of the immune response essential for host defense against pathogens. One group of clearly regulated IFNγ-induced genes is the GTPase family, which modulates survival of pathogens residing in phagosomes or vacuoles [39,40]. They are defined into three classes: Guanylate-binding proteins (Gbp's), the p47 GTPases and the Mx proteins. We found *Salmonella*-induced up-regulation of the first and second group, i.e. *Gbp1, Ifi47, Gbp2, Iigp *and *Irgm *(Table 2). IFNγ induces expression of p47 GTPases via activation of Stat1 which was also increased by *Salmonella *at days 2 and 3 p.i. Mutant mice with gene disruptions in IFNγ or Stat1 are significantly compromised in their immune response to microbial infections, including salmonellosis [41]. Thus the increased expression of IFNγ-related genes in colonic mucosa in the present study confirms the earlier proposed role of this cytokine in *Salmonella *infection.

### Pancreatits associated protein

The colonic mucosal gene most highly induced by *Salmonella *infection on the array was *Pap*, which was confirmed by Q-PCR of individual rat samples. PAP is a member of the Reg III gene family, which includes Regenerating islet-derived 3 gamma (*Reg3g*) which was also increased in our study. *Pap *expression is also increased in the rat ileal mucosa infected with *S. enteritidis *[3] and in the gastrointestinal tract of pigs infected with *Salmonella typimurium *[42]. Furthermore, significant up-regulation of intestinal mucosal *Pap *expression is described in IBD patients, whose bowel is chronically inflamed [43-45]. This suggests that PAP is a marker for acute as well as chronic inflammation. Biological functions of PAP in the intestine are not fully uncovered. Recently, it was proposed to function in innate immunity [44,46]. PAP was shown to have direct antimicrobial properties as it was able to bind and kill Gram-positive bacteria, but not Gram-negative *Salmonella typhimurium *[46]. Additional research will be needed to answer whether PAP is able to inhibit the growth of *Salmonella enteritidis. Pap *and *Reg3g *are expressed in several tissues and organs, but the small intestine has the highest expression under normal conditions. Only very low levels can be found in colon [47]. Indeed, *Pap *mRNA expression for most non-infected rats was below detection level (figure 4). Three rats did not express *Pap *at timepoint day 3 p.i. (figure 4), whereas at day 6 p.i. all rats expressed increased levels of *Pap*. Variation in infection kinetics between (outbred) rats is obviously reflected in *Pap *expression. We are currently investigating which mucosal cell types contain PAP and whether it is secreted to the intestinal lumen or to the serosal (blood) site. If secreted, PAP might be used as a non-specific marker to follow and quantify intestinal infection or inflammation in humans.

### Calprotectin

Calprotectin (*S100a8/a9*), a heterodimer of the two calcium-binding proteins S100A8 and S100A9, was up-regulated in the colonic mucosa by *Salmonella *(Table 2). Both subunits were increased in colon. Calprotectin is a 36 kDa calcium and zinc binding protein and constitutes approximately 60% of soluble cytosolic proteins in neutrophil granulocytes. Therefore, calprotectin is a marker of neutrophil influx and is elevated in a number of inflammatory conditions. In agreement with our results, Naughton et al (1996) also found increased levels of this marker in *Salmonella*-infected animals. Fecal calprotectin is emerging as a useful marker to quantify mucosal inflammation, not in the least because it appears to be stable in feces which can be obtained by non-invasive means [48].

### Differences between colon and ileum

Ileum and colon are both targets for *Salmonella*. Remarkably, the number of genes showing increased expression is larger than the number of genes showing decreased expression upon *Salmonella *infection in both ileum and colon. However, this is more extreme in colon than in ileum mucosa [5]. Technical bias is unlikely as in a flavonoid intervention study with rats and using the same array system and data handling the number of down-regulated genes was similar to the number of up-regulated genes [49]. In an *in vivo Salmonella *infection study in pigs only up-regulated and no down-regulated genes were observed [42].

The extent of the early response to *Salmonella *is similar for both intestinal segments: From all genes expressed above background level on the arrays, 0.21% of the genes expressed in the colon and 0.26% of genes expressed in the ileum [5] were affected at day 1 p.i. The colonic response is less than the ileal response at day 3 p.i., as 0.15% of colonic mucosal genes were affected versus 0.67% of ileal mucosal genes. The smaller colonic response could be due to differences in crypt-villus architecture of the ileal and colonic mucosa. Furthermore, the colonic mucosa, which is constitutively exposed to bacteria, might be more efficient in repressing host- or more specifically immunological responses to bacteria, including pathogens [50-52].

Ileum and colon show overlapping as well as distinct processes affected upon oral infection [5]. At early time points after oral infection i.e. transport processes and antimicrobial defenses were regulated in both intestinal segments, but the process-related genes did not fully overlap. At day 1 p.i., glucose transporters were increased in the ileum, whereas in colon ion transporters were induced. The role of ion transporters in water absorption support involvement of the distal part of the gut in diarrhea development during salmonellosis as reported earlier in humans [6]. The gene coding for antimicrobial defensin 5 was down-regulated by *Salmonella *in both ileum and colon. Other genes coding for antimicrobial proteins (*Pla2g2a *and lysozym) were clearly enhanced in the infected colon in contrast to ileal tissue [5]. At day 3 and 6 p.i. *Salmonella *reduced the expression of several phase I and II detoxification genes in the ileum, which was not observed in the colon. The downregulation of cytochrome P450 genes in ileum coincided with increased expression of inflammatory genes. It is known that inflammatory mediators can down-regulate cytochrome P450 genes [5,53,54]. This might suggest that the inflammatory response induced by *Salmonella *in colon, at later time-points, is smaller than in ileum. Nevertheless, both tissues showed signs of an inflammatory response at later time-points, but responsible genes were not the same. Mainly cytokines and chemokines were induced in the ileum, whereas in colon many interferon-related genes were up-regulated. No interferon response was observed in the ileum. Apparently, the immune response in the two intestinal segments is differentially regulated.

Finally, the *in vivo *transcriptional response of intact mucosa to invasion by *Salmonella *is represented by a limited number of regulated genes compared to *in vitro *studies with HT-29 cells [24]. *In vitro *models provide insight in complex mechanisms of *Salmonella*-host interaction. However, results should be interpreted with caution as *in vitro *systems show massive cell death at 24 hours, whereas only minor inflammatory changes are observed in the intestine 24 hours after infection with *Salmonella in vivo *[55]. Several genes like Toll like receptors, *Nf-κb *or *Il-8 *that are regulated by *Salmonella in vitro*, were not found to be regulated by *Salmonella *infection in the present *in vivo *study. Possibly, transcription of these genes is highly specific for particular cell types in the colonic mucosa. Identification of cell type-specific responses of potential target cells could be addressed *in vivo *using laser microdissection.

### FOS and mucosal barrier function

We consistently observed that diets supplemented with rapidly-fermentable prebiotics (such as FOS) increased translocation of *S. enteritidis *in rat infection studies despite stimulation of *Bifidobacteria *and *Lactobacilli *[23]. In other words, FOS decreases the resistance of the rat intestinal mucosa to intestinal pathogens. Because fermentation of FOS hardly occurs in the ileum of humans [14] and rats [15], it is unlikely that prebiotics facilitated translocation of *Salmonella *in the ileum. This is supported by the absence of ileal inflammation in FOS-fed and *Salmonella*-infected rats in contrast to profound cecal and colonic inflammation [11] The precise mechanism underlying the effects of FOS on the colon mucosa is not known. FOS itself, the changed intestinal microflora or its fermentation products (e.g. SCFA) could play a role. Prebiotics, such as FOS resist enzymatic hydrolysis by digestive enzymes secreted in the small intestine and reach the colon intact. The resident colonic microflora ferments these carbohydrates to lactic acid and short-chain fatty acids (SCFA). This results in lowering of the pH of intestinal contents and stimulation of e.g. *Bifidobacteria *and *Lactobacilli *[23,56]. These lactic acid bacteria are assumed to enhance resistance [12] but we found opposite effects [11,23]. As shown earlier, dietary FOS increases intestinal permeability in non-infected rats and even more in infected rats [13]. At present it is unknown whether intestinal permeability is increased in ileum or colon, nor whether it is induced by the presence of FOS or by its fermentation metabolites. It has been shown that SCFA can induce colonic mucosal injury and increase permeability [10]. Furthermore, *in vitro *studies showed that SCFA can enhance expression of virulence (e.g. invasion) genes of *Salmonella *typhimurium [57,58], but data on *in vivo *consequences have not been reported. Preliminary experiments of our lab showed no evidence for increased expression of virulence genes of *Salmonella enteritidis *in infected rats fed a FOS-diet (unpublished results).

From two independent rat infection studies we identified 26 colonic mucosal genes consistently affected by *Salmonella*. These 'robust' *Salmonella *target genes were all further induced by the FOS diet. The pronounced effects of FOS on *Salmonella *translocation were reflected by a modest but highly consistent increase of all *Salmonella *target genes. Moreover, the total number of genes induced by *Salmonella *is nearly 3 times higher in FOS-fed rats than in cellulose-fed rats. However, biological processes identified to be affected by *Salmonella *in colon of FOS-fed rats were not different from those observed in cellulose-fed rats. So, the quality of the colonic response was the same, but clearly the magnitude of the response was increased by FOS feeding. Based on the physiological effects, larger gene expression differences might have been expected. The modest responses observed might be due to our focus on *Salmonella*-induced gene expression. It can not be totally excluded that FOS targets other genes and processes related to barrier function (in absence of infection) than *Salmonella*. However, the genes affected by *Salmonella *in FOS-fed rats did not show involvement of additional processes in comparison to their cellulose-fed counterparts. In our view, the enhanced expression of colonic *Salmonella *target genes in FOS-fed animals concomitant with stimulated translocation of this invasive pathogen indicates that infection and related inflammation is worsened by FOS supplementation. Histological analyses of intestinal samples from previous FOS intervention studies of our lab did not show presence of intestinal mucosal inflammation in non-infected FOS-fed rats in contrast to post-infection samples (data not shown). Therefore, we feel that the observed aggravation of the intestinal response is due to interaction of FOS and *Salmonella*.

It should be stressed, that genes identified as *Salmonella *target genes in the present study are not necessarily *Salmonella *specific, but may well result from colonic inflammation in general and thus be similar in other enteric infections. Furthermore, effects of dietary FOS on gut barrier function may not be restricted to changes in mRNA expression, but exist on the translational or functional level of proteins. For instance, internalization of the tight-junction proteins occludin, claudin and junctional adhesion molecule-A, caused by IFNγ, results in profound mucosal barrier changes [59]. This cellular translocation can occur without concomitant changes in mRNA gene expression. Detection of such effects would require a different approach from transcriptomics. Many studies report on possible therapeutic effects of FOS on intestinal disease such as IBD and pathogenic infection. In addition to an increase in ''beneficial" bacteria, the potential beneficial effects of FOS are based on the effects on surrogate markers, e.g. increase of mucin production [60], increase of the size and cytokine production of Peyer's patches and increased faecal or ileal IgA [61-63]. Changes in these markers are often presumed to reflect increased barrier function or resistance to pathogenic bacteria, but concomitant actual measurements of these functional effects are missing. In our study, genes involved in antimicrobial defense, immune response and inflammation were all induced by *Salmonella *infection and further enhanced by dietary FOS, but concomitantly translocation of *Salmonella *was evident and stimulated by FOS. Therefore, these surrogate markers should be interpreted with caution and always correlated with functional effects or clinical endpoints.

In this study we compared *Salmonella-*induced gene expression changes of two independent rat infection experiments at early time points after oral infection. The gene expression results were analyzed at two levels, at the level of gene expression itself and at the level of biological processes. Analysis at the level of gene expression showed some variation in the expression of individual genes between the two studies (table 2, Additional File 3). This variation between two studies can be due to the different time-points studied and the use of outbred rats showing inter-individual differences in infection kinetics. Rats did not all respond to *Salmonella *at the same time p.i. which is e.g. shown by individual gene expression levels of PAP (figure 4) and by serum IFNγ levels (figure 3). Variation in infection kinetics and inter-individual variation are expected features of infection studies in outbred species [64]. It can be argued that differences between studies, due to differences in time points measured or infection kinetics, will result in more pronounced variance at the level of individual genes than at the level of physiological processes [65,66]. Indeed, analysis of gene expression at the level of biological processes showed that both studies gave highly comparable *Salmonella*-induced effects at early time points.

In the dietary infection study, we were interested whether the FOS-stimulated *Salmonella *translocation was reflected in colonic gene expression changes. We observed an overlap in gene expression changes observed in the two experimental diets, and an additional set of 58 genes which were only significantly affected in the FOS-fed rats. Although the list of altered genes was different in FOS fed rats, this was not the case at the process level as exactly the same processes were observed for both dietary groups. This indicates involvement of similar underlying biological processes in cellulose and FOS-fed infected rats and no obvious role for other processes.

Comparison at the level of biological processes is a powerful tool to interpret microarray experiments and enables comparison of different microarray datasets [67]. Comparison at gene level has some drawbacks, one is redundancy in gene function, which means that different genes can provide the same physiological effect. In addition, the homeostatic condition as well as the precise nature of the stimulus will determine how individual genes within a process are controlled to provide the necessary physiological response. Differences in responses of individual genes are filtered out when they are analyzed at the level of pathways or processes. However, the translation of differentially expressed genes into biological processes also suffers from limitations [68]. The most important limitation is that annotations to pathways and processes are incomplete. Therefore it is important that results from pathway analysis are manually supplemented with the remaining significant genes using biological databases and scientific literature.

## Conclusion

In conclusion, our results show that, in addition to the ileum, the colon mucosa is clearly a target for *Salmonella *infection. Early *Salmonella*-induced changes were observed in transport and oxidative stress, while at later stage, most likely secondary, infection and inflammation responses were observed. Some findings confirm expected results, such as induction of an immune and inflammatory response. However, the *Salmonella*-induced immune response in colon is clearly different from that in ileum. We newly identified that colonic transport processes and proteolysis are affected by *Salmonella *infection and that pancreatitis associated protein was the most responsive gene in *Salmonella *infected rat colon.

An important observation is that FOS-stimulated *Salmonella *translocation (as measured by urinary NO_x_), does not induce other processes than those observed in cellulose-fed and *Salmonella *infected rats. So, the quality or diversity of the colonic host response to *Salmonella *is not affected by colonic FOS fermentation in contrast to the magnitude of response. As far as we know, there are no literature data pointing to a functional effect of FOS in the ileum. Therefore, the FOS effects on *Salmonella *translocation are most likely due to colonic effects. Understanding the changes caused by FOS alone may provide insight in processes that ultimately result in the observed weakening of the barrier.

## Methods

### Time course infection study

#### Animals, diet and infection

The experimental protocols were approved by the animal welfare committee of Wageningen University (Wageningen, the Netherlands). Specific pathogen-free male outbred 9 weeks old Wister rats (WU, Harlan, Horst, the Netherlands, n = 48 in total), were housed individually in metabolic cages. All animals were kept in a temperature (22–24°C) and humidity (50–60%) controlled room with a 12 h light/dark cycle (lights on from 6 AM to 6 PM). Rats were fed a purified diet during the whole experimental period. The diet contained (per kg) 200 g acid casein, 502 g glucose, 160 g palm oil, 40 g corn oil, 50 g cellulose, 35 g mineral mix (without calcium) and 10 g vitamin mix according to AIN93 recommendations [69]. Diets were low in calcium content (20 mmol CaHPO_4_.2H_2_O/kg) and high in fat content (200 g fat/kg)[16] to mimic the composition of a Western human diet. Food and demineralized drinking water were supplied *ad libitum*. The animals were acclimatized to the housing and dietary condition for 11 days, after which they were orally infected with *S. enteritidis *(clinical isolate, phage type 4 according to international standards; B1214 culture of NIZO food research, Ede, the Netherlands). *Salmonella *infection was performed by gastric gavage with 1 mL of saline containing 3 × 10^9 ^colony forming units (CFU) of *S. enteritidis*. Non-infected rats received saline only (control). *S. enteritidis *was cultured and stored, as described earlier [23]. Fresh fecal samples were collected on days 1, 2, 3 and 6 post infection (p.i.) and analyzed for viable *Salmonella *by plating 10-fold dilutions in sterile saline on Modified Brilliant Green Agar (Oxoid, Basingstoke, UK) and incubating aerobically overnight at 37°C. Sulphamandelate (Oxoid) was added to the agar plates to suppress swarming bacteria, such as Proteus species. The detection limit of this method was 10^2 ^CFU/g fecal wet weight. Total 24 h urine samples were collected from the day before oral infection of the rats until day 6 after infection. Urines were preserved by adding oxytetracycline to the urine collection vessels of the metabolic cages, and analyzed for the nitric oxide metabolites nitrite and nitrate (summed as NO_x_) by a colorimetric method (Nr. 1746081; Roche diagnostics, Mannheim, Germany).

Rats were sacrificed on day 1, 3 or 6 post infection and control (n = 8 rats per treatment and per time point). Rats were killed by carbon dioxide inhalation. Blood was collected by orbita puncture. Blood was coagulated for 30 minutes at room temperature, cooled to 4°C and centrifuged 20 minutes by 3000 g. Serum was collected and frozen at -80°C. The mesenteric lymph nodes (MLN), spleen and liver were excised aseptically, weighed, homogenized (Ultraturrax Pro200, Pro Scientific Inc. Oxford, CT) in sterile saline, serially diluted, and plated to culture for *Salmonella*, as described above. The detection limit was 10^2 ^CFU/g tissue. To obtain colonic mucosa, the colon was taken out, longitudinally opened and colonic contents removed by a quick rinse in 154 mM KCl. The mucosa was scraped off using a spatula. The scrapings were immediately frozen in liquid nitrogen and stored at -80°C for RNA extraction.

#### RNA isolation

Colon scrapings were homogenized in liquid N_2 _using a mortar and pestle cooled with liquid N_2._(Fisher Emergo, Landsmeer, The Netherlands). Total RNA was isolated from these homogenates using TRIzol reagent (Invitrogen, San Diego, CA) according to the manufacturer's instructions. Total RNA was purified using Rneasy columns (Qiagen, Chatsworth, CA). Absence of RNA degradation was checked on a 1% TBE/agarose gel after 1 hour incubation at 37°C. The purity and concentration were measured with the Nanodrop (Isogen Life Science, Maarssen, The Netherlands). OD A_260_/A_280 _ratios were all between 2.08 and 2.10 indicating RNA of high purity.

#### Analysis of mRNA expression by Oligo Arrays

For microarray hybridization, equal amounts of RNA of each animal were pooled per treatment group. Arrays were performed in duplicate. For this, RNA pools were split and separately reverse transcribed and labeled with Cy-5. A standard reference sample, consisting of a pool of all colonic RNA was labeled with Cy-3. For each oligo array, 35 μg of total RNA was used to make Cy-5 or Cy-3 labeled cDNA. Total RNA was mixed with 4 μg T21 primer, heated at 65°C for 3 min (RNA denaturation) followed by 25°C for 10 min (primer annealing). cDNA was synthesized by adding 5× first strand buffer (Invitrogen), 10 mM DTT, 0.5 mM dATP, 0.5 mM dGTP, 0.5 mM dTTP, 0.04 mM dCTP, 0.04 mM Cy5-dCTP or Cy3-dCTP, 1.2 U RnaseOUT and 6 U SuperScript II Reverse Transcriptase to a total volume of 62.5 μL. The reaction was incubated at 42°C for 2 h. Purification, precipitation and denaturation of the labeled cDNA were performed as described earlier [70].

The 44 K rat whole genome Agilent array (G4131A, Agilent Technologies, Inc. Santa Clara, CA) used consists of 44290 60-mer rat oligonucleotides, including ~ 3000 control spots. The Cy5 labeled cDNAs of the *Salmonella *infected groups and the non-infected groups were mixed 1:1 with the Cy3 reference labeled cDNA, mixed with 2× hybridization buffer (Agilent Technologies) and 10× control targets (Agilent Technologies) and hybridized for 17 hours at 60°C in Agilent hybridization chambers in an Agilent hybridization oven rotating at 4 rpm (Agilent Technologies). After hybridization the arrays were washed with an SSPE wash procedure (Agilent Technologies) and scanned with an Agilent Microarray Scanner (Agilent Technologies).

#### Data analysis

Signal intensities for each spot were quantified using Feature Extraction 8.1 (Agilent Technologies). The data of the time course infections study are available in Additional File 4 and have been deposited in NCBIs Gene Expression Omnibus [71] and are accessible through GEO Series accession number GSE7496. Median density values and background values of each spot were extracted for both the experimental samples (Cy5) and the reference samples (Cy3). Quality check was performed for each microarray using both LimmaGUI package in R from Bioconductor [72] and Microsoft Excel. Data was exported into GeneMaths XT (Applied Maths, Sint-Martens-Latem, Belgium) for analysis. We discarded spots with an average intensity, over all arrays, of Cy5 lower than 2-fold above average background. Then, the Cy5 intensities were normalized against the Cy3 reference as described before [73]. The gene expressions of duplicate arrays were averaged. Array data of non-infected rats, killed on section day 1 and 6 were highly comparable and could therefore be considered as one group and were averaged. For unknown reason, arrays of non-infected rats killed on day 3 showed reduced expression of 14 mast cell protease genes when compared with non-infected rats of both days 1 and 6, which were highly comparable. Therefore, we decided not to include the non-infected rats of day 3. Cluster analysis and Principle component analysis were performed using GeneMaths XT. Infected/control ratio's between 0–1 were expressed as the negative inverse (-1/value) for easier interpretation. Genes that changed more than 2-fold in comparison with controls at one of the time points studied were selected for pathway analysis. Pathway analysis was performed using two pathway programs, MetaCore (GeneGo Inc, St. Joseph, MI)[18]and ErmineJ [19], using Agilent gene annotation (Agilent Technologies, version 20060331). Processes were identified using statistical over-representation in both pathway programs. Since only 40% of the genes were annotated to GO processes in both pathway programs, processes with a p-value < 0.001 were manually supplemented with non-annotated genes with FC > 2 using biological databases (BIOcarta, SOURCE, GenMAPP, KEGG) and scientific literature.

#### Analysis of mRNA expression by Real-time Quantitative RT-PCR

Real-time Quantitative RT-PCR (Q-PCR) was performed on individual samples (n = 8 per group). 1 μg of RNA of all individual samples was used for the cDNA synthesis using the iScript cDNA synthesis kit of Bio-Rad Laboratories (Veenendaal, The Netherlands). Real-time reactions were performed by means of the iQ SYBR Green Supermix of Bio-Rad using the MyIQ single-color real-time PCR detection system (Bio-Rad). Each reaction (25 μl) contained 12.5 μl iQ SYBR green supermix, 1 μl forward primer (10 μM), 1 μl reverse primer (10 μM), 8.5 μl RNase-free water and 2 μl diluted cDNA. The following cycles were performed 1× 3 min at 95°C, 40 amplification cycles (40× 10 s 95°C, 45 s 60°C), 1× 1 min 95°C, 1× 1 min 62°C and a melting curve (80× 10 s 55°C with an increase of 0.5°C per 10 s). A negative control without cDNA template was run with every assay. The optimal melting point of dsDNA (Tm) and the efficiency of the reaction were optimized beforehand. Data were normalized against the reference genes Ribosomal protein S29 (*Rps29*), ADP-Ribosylation Factor 1 (*Arf1*) and β-actin. *Rps29 *and *Arf1 *were chosen on the basis of microarray data which showed similar expression levels for all microarrays, β-actin was chosen as this is a well accepted reference gene. Primers were designed using Beacon designer 4 (Premier Biosoft International, Palo Alto, CA). For sequences see Additional File 5. A standard curve for all genes including reference genes was generated using serial dilutions of a pooled sample (cDNA from all reactions). mRNA levels were determined from the appropriate standard curve. Samples with mRNA levels below the lowest standard value, and thus below detection level, were given half the value of this lowest standard. Analysis of all individual samples was performed in duplicate.

#### Serum Interferon Gamma

The serum Interferon Gamma (IFNγ) concentration of individual rats was determined by an enzyme-linked immunosorbent assay (ELISA) specific for rats (Biosource International, Camarillo, CA) according to the manufacturer's protocol.

### Dietary infection study

#### Animals, diet and infection

A dietary intervention was performed to study the effect of FOS on *S. enteritidis*-induced gene expression. Specific pathogen-free male outbred Wister rats (8 weeks old, mean body weight of 253 g; n = 48 in total) were housed as described above (time course infection study). Rats were fed the same diet as described above. The experimental diets both contained 20 g/kg cellulose and were supplemented with either 60 g/kg FOS (purity 93%; Raftilose P95, Orafti, Tienen, Belgium) or additional 60 g/kg cellulose as described earlier [23]. Animals were fed restricted quantities (14 g/day) of the purified diet. Restricted food intake was necessary to prevent differences in food consumption and hence differences in vitamin and mineral intake as observed earlier in FOS interventions [11]. After an adaptation period of 14 days, rats were orally infected with 4 × 10^8 ^CFU of *S. enteritidis *or control-treated as described above. On day 2 p.i., 12 infected FOS-fed rats, 12 infected rats fed the cellulose diet, and 12 control-treated non-infected rats fed the cellulose diet were sacrificed to obtain colonic mucosal RNA. Two additional groups of rats fed either FOS (n = 6) or the cellulose diet (n = 6) and infected with *Salmonella *were kept until day 8 p.i. for determination of urinary NO_x _excretion in time as described above.

#### Analysis of mRNA expression by Oligo Arrays and Real-time Quantitative RT-PCR

RNA isolation and analysis of mRNA expression by microarray (pooled samples) and Q-PCR (n = 12 per treatment group) were performed as described above. Arrays were scanned with a Scanarray Express HT scanner (Perkin Elmer). Signal intensities for each spot were quantified using ArrayVision 8.0 (GE Healthcare life sciences). Data analysis was performed as described above. The data of the dietary infection study available in Additional File 6 and have been deposited in NCBIs Gene Expression Omnibus [71] and are accessible through GEO Series accession number GSE7472.

### Statistical analysis

Results are expressed as median or mean depending on normality of distribution as indicated. We used Prism 4 for all statistics (Prism 4, GraphPad software Inc., San Diego, CA). Data was analyzed using the Student's t-test (two-sided). Non-normally distributed data was analyzed using the non-parametric Mann-Whitney U test (two sided). Differences were considered statistically significant when p < 0.05.

## Authors' contributions

WR, EK, CV and SR performed the experiments. WR performed the data analysis, interpreted the results and drafted the manuscript. JK and IB participated in the design of the study, in evaluation of the results and in revision of the manuscript. RvdM and MK discussed the results and critically read the manuscript. All authors read and approved the final manuscript.

## Supplementary Material

Additional file 1 Salmonella affected colonic genes. Genes that were upregulated or downregulated at least 1.5-fold in rat colon mucosa by *Salmonella *at days 1, 3 and 6 after oral *Salmonella *infection compared to colon mucosa of non-infected rats. Genes are ordered based on function and within functional category based on absolute fold change. Click here for file

Additional file 2 Housekeeping and cell type specific genes. The fold change in expression of housekeeping genes and cell-type specific genes in the colon mucosa at days 1, 3 and 6 after oral *Salmonella *infection. Click here for file

Additional file 3 Dietary modulated genes. Processes regulated in colon by *Salmonella *at day 2 in cellulose fed and FOS fed rats. Click here for file

Additional file 4 Time course infection study. Complete dataset of the time course infection study. Click here for file

Additional file 5 Primer sequences. Sequences of the primers used for Q-PCR analysis. Click here for file

Additional file 6 Dietary infection study. Complete dataset of the dietary infection study. Click here for file

## References

[B1] Barrow PA (2007). Salmonella infections: immune and non-immune protection with vaccines. Avian Pathol.

[B2] Herikstad H, Motarjemi Y, Tauxe RV (2002). Salmonella surveillance: a global survey of public health serotyping. Epidemiol Infect.

[B3] Havelaar AH, Garssen J, Takumi K, Koedam MA, Dufrenne JB, van Leusden FM, de La Fonteyne L, Bousema JT, Vos JG (2001). A rat model for dose-response relationships of Salmonella Enteritidis infection. J Appl Microbiol.

[B4] Naughton PJ, Grant G, Spencer RJ, Bardocz S, Pusztai A (1996). A rat model of infection by Salmonella typhimurium or Salm. enteritidis. J Appl Bacteriol.

[B5] Rodenburg W, Bovee-Oudenhoven IMJ, Kramer E, van der Meer R, Keijer J (2007). Gene expression response of the rat small intestine following oral Salmonella infection. Physiological Genomics.

[B6] Mandal BK, Mani V (1976). Colonic involvement in salmonellosis. Lancet.

[B7] McGovern VJ, Slavutin LJ (1979). Pathology of salmonella colitis. Am J Surg Pathol.

[B8] Vender RJ, Marignani P (1983). Salmonella colitis presenting as a segmental colitis resembling Crohn's disease. Dig Dis Sci.

[B9] Honer zu Bentrup K, Ramamurthy R, Ott CM, Emami K, Nelman-Gonzalez M, Wilson JW, Richter EG, Goodwin TJ, Alexander JS, Pierson DL, Pellis N, Buchanan KL, Nickerson CA (2006). Three-dimensional organotypic models of human colonic epithelium to study the early stages of enteric salmonellosis. Microbes Infect.

[B10] Argenzio RA, Meuten DJ (1991). Short-chain fatty acids induce reversible injury of porcine colon. Dig Dis Sci.

[B11] Bovee-Oudenhoven IM, ten Bruggencate SJ, Lettink-Wissink ML, van der Meer R (2003). Dietary fructo-oligosaccharides and lactulose inhibit intestinal colonisation but stimulate translocation of salmonella in rats. Gut.

[B12] Gibson GR (1999). Dietary modulation of the human gut microflora using the prebiotics oligofructose and inulin. J Nutr.

[B13] Ten Bruggencate SJ, Bovee-Oudenhoven IM, Lettink-Wissink ML, Van der Meer R (2005). Dietary fructooligosaccharides increase intestinal permeability in rats. J Nutr.

[B14] Andersson HB, Ellegard LH, Bosaeus IG (1999). Nondigestibility characteristics of inulin and oligofructose in humans. J Nutr.

[B15] Heijnen AM, Brink EJ, Lemmens AG, Beynen AC (1993). Ileal pH and apparent absorption of magnesium in rats fed on diets containing either lactose or lactulose. Br J Nutr.

[B16] Bovee-Oudenhoven IM, Termont DS, Weerkamp AH, Faassen-Peters MA, Van der Meer R (1997). Dietary calcium inhibits the intestinal colonization and translocation of Salmonella in rats. Gastroenterology.

[B17] Oudenhoven IM, Klaasen HL, Lapre JA, Weerkamp AH, Van der Meer R (1994). Nitric oxide-derived urinary nitrate as a marker of intestinal bacterial translocation in rats. Gastroenterology.

[B18] Ekins S, Nikolsky Y, Bugrim A, Kirillov E, Nikolskaya T (2007). Pathway mapping tools for analysis of high content data. Methods Mol Biol.

[B19] Lee HK, Braynen W, Keshav K, Pavlidis P (2005). ErmineJ: tool for functional analysis of gene expression data sets. BMC Bioinformatics.

[B20] Hsiao LL, Dangond F, Yoshida T, Hong R, Jensen RV, Misra J, Dillon W, Lee KF, Clark KE, Haverty P, Weng Z, Mutter GL, Frosch MP, Macdonald ME, Milford EL, Crum CP, Bueno R, Pratt RE, Mahadevappa M, Warrington JA, Stephanopoulos G, Stephanopoulos G, Gullans SR (2001). A compendium of gene expression in normal human tissues. Physiol Genomics.

[B21] Knight PA, Pemberton AD, Robertson KA, Roy DJ, Wright SH, Miller HR (2004). Expression profiling reveals novel innate and inflammatory responses in the jejunal epithelial compartment during infection with Trichinella spiralis. Infect Immun.

[B22] Anderle P, Rakhmanova V, Woodford K, Zerangue N, Sadee W (2003). Messenger RNA expression of transporter and ion channel genes in undifferentiated and differentiated Caco-2 cells compared to human intestines. Pharm Res.

[B23] Ten Bruggencate SJ, Bovee-Oudenhoven IM, Lettink-Wissink ML, Van der Meer R (2003). Dietary fructo-oligosaccharides dose-dependently increase translocation of salmonella in rats. J Nutr.

[B24] Eckmann L, Smith JR, Housley MP, Dwinell MB, Kagnoff MF (2000). Analysis by high density cDNA arrays of altered gene expression in human intestinal epithelial cells in response to infection with the invasive enteric bacteria Salmonella. J Biol Chem.

[B25] Kagnoff MF, Eckmann L (1997). Epithelial cells as sensors for microbial infection. J Clin Invest.

[B26] Naughton PJ, Grant G, Ewen SW, Spencer RJ, Brown DS, Pusztai A, Bardocz S (1995). Salmonella typhimurium and Salmonella enteritidis induce gut growth and increase the polyamine content of the rat small intestine in vivo. FEMS Immunol Med Microbiol.

[B27] Gray JT, Fedorka-Cray PJ, Stabel TJ, Ackermann MR (1995). Influence of inoculation route on the carrier state of Salmonella choleraesuis in swine. Vet Microbiol.

[B28] Rout WR, Formal SB, Dammin GJ, Giannella RA (1974). Pathophysiology of Salmonella diarrhea in the Rhesus monkey: Intestinal transport, morphological and bacteriological studies. Gastroenterology.

[B29] Hughes EA, Galan JE (2002). Immune response to Salmonella: location, location, location?. Immunity.

[B30] Kops SK, Lowe DK, Bement WM, West AB (1996). Migration of Salmonella typhi through intestinal epithelial monolayers: an in vitro study. Microbiol Immunol.

[B31] Chong C, Bost KL, Clements JD (1996). Differential production of interleukin-12 mRNA by murine macrophages in response to viable or killed Salmonella spp. Infect Immun.

[B32] Eckmann L, Kagnoff MF (2001). Cytokines in host defense against Salmonella. Microbes Infect.

[B33] John B, Rajagopal D, Pashine A, Rath S, George A, Bal V (2002). Role of IL-12-independent and IL-12-dependent pathways in regulating generation of the IFN-gamma component of T cell responses to Salmonella typhimurium. J Immunol.

[B34] Jouanguy E, Doffinger R, Dupuis S, Pallier A, Altare F, Casanova JL (1999). IL-12 and IFN-gamma in host defense against mycobacteria and salmonella in mice and men. Curr Opin Immunol.

[B35] Mastroeni P, Harrison JA, Chabalgoity JA, Hormaeche CE (1996). Effect of interleukin 12 neutralization on host resistance and gamma interferon production in mouse typhoid. Infect Immun.

[B36] Mastroeni P, Harrison JA, Robinson JH, Clare S, Khan S, Maskell DJ, Dougan G, Hormaeche CE (1998). Interleukin-12 is required for control of the growth of attenuated aromatic-compound-dependent salmonellae in BALB/c mice: role of gamma interferon and macrophage activation. Infect Immun.

[B37] VanCott JL, Staats HF, Pascual DW, Roberts M, Chatfield SN, Yamamoto M, Coste M, Carter PB, Kiyono H, McGhee JR (1996). Regulation of mucosal and systemic antibody responses by T helper cell subsets, macrophages, and derived cytokines following oral immunization with live recombinant Salmonella. J Immunol.

[B38] Kagaya K, Watanabe K, Fukazawa Y (1989). Capacity of recombinant gamma interferon to activate macrophages for Salmonella-killing activity. Infect Immun.

[B39] Taylor GA, Feng CG, Sher A (2004). p47 GTPases: regulators of immunity to intracellular pathogens. Nat Rev Immunol.

[B40] Singh SB, Davis AS, Taylor GA, Deretic V (2006). Human IRGM induces autophagy to eliminate intracellular mycobacteria. Science.

[B41] Mastroeni P, Clare S, Khan S, Harrison JA, Hormaeche CE, Okamura H, Kurimoto M, Dougan G (1999). Interleukin 18 contributes to host resistance and gamma interferon production in mice infected with virulent Salmonella typhimurium. Infect Immun.

[B42] Niewold TA, Veldhuizen EJ, van der Meulen J, Haagsman HP, de Wit AA, Smits MA, Tersteeg MH, Hulst MM (2007). The early transcriptional response of pig small intestinal mucosa to invasion by Salmonella enterica serovar typhimurium DT104. Mol Immunol.

[B43] Dieckgraefe BK, Stenson WF, Korzenik JR, Swanson PE, Harrington CA (2000). Analysis of mucosal gene expression in inflammatory bowel disease by parallel oligonucleotide arrays. Physiol Genomics.

[B44] Gironella M, Iovanna JL, Sans M, Gil F, Penalva M, Closa D, Miquel R, Pique JM, Panes J (2005). Anti-inflammatory effects of pancreatitis associated protein in inflammatory bowel disease. Gut.

[B45] Ogawa H, Fukushima K, Naito H, Funayama Y, Unno M, Takahashi K, Kitayama T, Matsuno S, Ohtani H, Takasawa S, Okamoto H, Sasaki I (2003). Increased expression of HIP/PAP and regenerating gene III in human inflammatory bowel disease and a murine bacterial reconstitution model. Inflamm Bowel Dis.

[B46] Cash HL, Whitham CV, Behrendt CL, Hooper LV (2006). Symbiotic bacteria direct expression of an intestinal bactericidal lectin. Science.

[B47] Iovanna JL, Keim V, Bosshard A, Orelle B, Frigerio JM, Dusetti N, Dagorn JC (1993). PAP, a pancreatic secretory protein induced during acute pancreatitis, is expressed in rat intestine. Am J Physiol.

[B48] Poullis A, Foster R, Northfield TC, Mendall MA (2002). Review article: faecal markers in the assessment of activity in inflammatory bowel disease. Aliment Pharmacol Ther.

[B49] de Boer VC, van Schothorst EM, Dihal AA, van der Woude H, Arts IC, Rietjens IM, Hollman PC, Keijer J (2006). Chronic quercetin exposure affects fatty acid catabolism in rat lung. Cell Mol Life Sci.

[B50] Collier-Hyams LS, Sloane V, Batten BC, Neish AS (2005). Cutting edge: bacterial modulation of epithelial signaling via changes in neddylation of cullin-1. J Immunol.

[B51] Ismail AS, Hooper LV (2005). Epithelial cells and their neighbors. IV. Bacterial contributions to intestinal epithelial barrier integrity. Am J Physiol Gastrointest Liver Physiol.

[B52] Kelly D, Campbell JI, King TP, Grant G, Jansson EA, Coutts AG, Pettersson S, Conway S (2004). Commensal anaerobic gut bacteria attenuate inflammation by regulating nuclear-cytoplasmic shuttling of PPAR-gamma and RelA. Nat Immunol.

[B53] Morgan ET, Li-Masters T, Cheng PY (2002). Mechanisms of cytochrome P450 regulation by inflammatory mediators. Toxicology.

[B54] Renton KW (2001). Alteration of drug biotransformation and elimination during infection and inflammation. Pharmacol Ther.

[B55] Finlay BB, Brumell JH (2000). Salmonella interactions with host cells: in vitro to in vivo. Philos Trans R Soc Lond B Biol Sci.

[B56] Ten Bruggencate SJ, Bovee-Oudenhoven IM, Lettink-Wissink ML, Katan MB, van der Meer R (2006). Dietary fructooligosaccharides affect intestinal barrier function in healthy men. J Nutr.

[B57] Durant JA, Corrier DE, Ricke SC (2000). Short-chain volatile fatty acids modulate the expression of the hilA and invF genes of Salmonella typhimurium. J Food Prot.

[B58] Lawhon SD, Maurer R, Suyemoto M, Altier C (2002). Intestinal short-chain fatty acids alter Salmonella typhimurium invasion gene expression and virulence through BarA/SirA. Mol Microbiol.

[B59] Bruewer M, Utech M, Ivanov AI, Hopkins AM, Parkos CA, Nusrat A (2005). Interferon-gamma induces internalization of epithelial tight junction proteins via a macropinocytosis-like process. Faseb J.

[B60] Schmidt-Wittig U, Enss ML, Coenen M, Gartner K, Hedrich HJ (1996). Response of rat colonic mucosa to a high fiber diet. Ann Nutr Metab.

[B61] Hosono A, Ozawa A, Kato R, Ohnishi Y, Nakanishi Y, Kimura T, Nakamura R (2003). Dietary fructooligosaccharides induce immunoregulation of intestinal IgA secretion by murine Peyer's patch cells. Biosci Biotechnol Biochem.

[B62] Roller M, Rechkemmer G, Watzl B (2004). Prebiotic inulin enriched with oligofructose in combination with the probiotics Lactobacillus rhamnosus and Bifidobacterium lactis modulates intestinal immune functions in rats. J Nutr.

[B63] Swanson KS, Grieshop CM, Flickinger EA, Bauer LL, Healy HP, Dawson KA, Merchen NR, Fahey GC (2002). Supplemental fructooligosaccharides and mannanoligosaccharides influence immune function, ileal and total tract nutrient digestibilities, microbial populations and concentrations of protein catabolites in the large bowel of dogs. J Nutr.

[B64] Hyland KA, Kohrt L, Vulchanova L, Murtaugh MP (2006). Mucosal innate immune response to intragastric infection by Salmonella enterica serovar Choleraesuis. Mol Immunol.

[B65] Segal E, Friedman N, Kaminski N, Regev A, Koller D (2005). From signatures to models: understanding cancer using microarrays. Nat Genet.

[B66] Subramanian A, Tamayo P, Mootha VK, Mukherjee S, Ebert BL, Gillette MA, Paulovich A, Pomeroy SL, Golub TR, Lander ES, Mesirov JP (2005). Gene set enrichment analysis: a knowledge-based approach for interpreting genome-wide expression profiles. Proc Natl Acad Sci U S A.

[B67] Manoli T, Gretz N, Grone HJ, Kenzelmann M, Eils R, Brors B (2006). Group testing for pathway analysis improves comparability of different microarray datasets. Bioinformatics.

[B68] Khatri P, Draghici S (2005). Ontological analysis of gene expression data: current tools, limitations, and open problems. Bioinformatics.

[B69] Reeves PG, Nielsen FH, Fahey GC (1993). AIN-93 purified diets for laboratory rodents: final report of the American Institute of Nutrition ad hoc writing committee on the reformulation of the AIN-76A rodent diet. J Nutr.

[B70] van Hal NL, Vorst O, van Houwelingen AM, Kok EJ, Peijnenburg A, Aharoni A, van Tunen AJ, Keijer J (2000). The application of DNA microarrays in gene expression analysis. J Biotechnol.

[B71] NCBI GEO website http://www.ncbi.nlm.nih.gov/geo/.

[B72] Wettenhall JM, Smyth GK (2004). limmaGUI: a graphical user interface for linear modeling of microarray data. Bioinformatics.

[B73] Pellis L, Franssen-van Hal NL, Burema J, Keijer J (2003). The intraclass correlation coefficient applied for evaluation of data correction, labeling methods, and rectal biopsy sampling in DNA microarray experiments. Physiol Genomics.

