# Supplementary material for: Salmonella induces prominent gene expression in the rat colon
Source: BMC Microbiol. 2007 Sep 12;7:84. doi: 10.1186/1471-2180-7-84 (PMC2048963; doi:10.1186/1471-2180-7-84)
Supplement: Additional file 1 — Salmonella affected colonic genes. Genes that were upregulated or downregulated at least 1.5-fold in rat colon mucosa by Salmonella at days 1, 3 and 6 after oral Salmonella infection compared to colon mucosa of non-infected rats. Genes are ordered based on function and within functional category based on absolute fold change. [file 1471-2180-7-84-S1.doc]

Additional file 1

Genes that were upregulated or downregulated at least 1.5-fold in rat colon mucosa by *Salmonella* at days 1, 3 and 6 after oral *Salmonella* infection compared to colon mucosa of non-infected rats. Genes are ordered based on function and within functional category based on absolute fold change. Values in bold exceed cut-off value FC>2 or FC<-2. Values -1,5 <FC< 1,5 are indicated by (-).

|  |  |  | **Fold Change infected vs non-infected rats on different days p.i.** | | |
| --- | --- | --- | --- | --- | --- |
| **Gene Name** | **Gene symbol** | **Sequence ID** |
|  |  |  | **Day 1** | **Day 3** | **Day 6** |
| **Transport** |  |  |  |  |  |
| chloride channel calcium activated 6 | *Clca6* | NM_201419 | **2,3** | **2,2** | **3,7** |
| calcium channel, voltage-dependent, alpha 1I subunit | *Cacna1i* | NM_020084 | **2,2** | 1,5 | **-** |
| solute carrier family 4, member 1 | *Slc4a1* | NM_012651 | **2,0** | 1,7 | **-** |
| ATPase, H+/K+ transporting, nongastric, alpha polypeptide | *Atp12a* | NM_133517 | **2,8** | **-** | **2,0** |
| transporter 1, ATP-binding cassette, sub-family B | *MDR/Tap1* | NM_032055 | - | 1,8 | **2,8** |
| solute carrier family 20 (phosphate transporter), member 1 | *Slc20a1* | NM_031148 | **2,1** | **-** | **-** |
| solute carrier family 15 (oligopeptide transporter), member 1 | *Slc15a1* | NM_057121 | **2,0** | 1,5 | 1,6 |
| **Oxidative stress** |  |  |  |  |  |
| dual oxidase 2 | *Duox2* | NM_024141 | 1,9 | **2,4** | **2,8** |
| glutathione peroxidase 2 | *Gpx2* | NM_183403 | - | **2,3** | **3,0** |
| xanthine dehydrogenase | *Xdh* | NM_017154 | - | 1,8 | **2,5** |
| **Immune response, Antimicrobial defense and Inflammatory response** |  |  |  |  |  |
| pancreatitis-associated protein | *Pap* | NM_053289 | **11,4** | **44,6** | **114,3** |
| phospholipase A2, group IIA | *Pla2g2a* | NM_031598 | **3,4** | **5,2** | **10,5** |
| Rat class III Fc-gamma receptor mRNA, complete cds. | *Fcgr3* | M64368 | **2,1** | 1,6 | - |
| immunoglobulin superfamily, member 4 | *Igsf4d* | XM_340958 | **2,1** | 1,5 | - |
| colony stimulating factor 2 (granulocyte-macrophage) | *Csf2* | XM_340799 | **2,0** | 1,6 | - |
| Rat MHC class I truncated cell surface antigen mRNA. | *RT1-Aw2* | M10094 | **2,0** | - | 1,9 |
| Interleukin enhancer-binding factor 1 |  | XM_221212 | **2,0** | 1,5 | - |
| Defensin 5 precursor |  | XM_214386 | -1,9 | -1,6 | -1,6 |
| tissue-type transglutaminase | *Tgm2* | NM_019386 | - | **2,3** | **4,9** |
| regenerating islet-derived 3 gamma | *Reg3g* | NM_173097 | - | **2,3** | **4,2** |
| interleukin 1 beta | *Il1b* | NM_031512 | **-** | **2,1** | **4,0** |
| interleukin 1 alpha | *Il1a* | NM_017019 | 1,8 | **2,0** | **2,3** |
| TRAF2 binding protein | *TRAF2* | XM_227712 | 1,8 | **2,8** | **4,4** |
| lysozyme | *Lyz* | NM_012771 | **-** | **-** | **5,4** |
| nitric oxide synthase 2, inducible | *Nos2* | NM_012611 | - | 1,6 | **4,0** |
| Ly6-C antigen gene | *Ly6c* | NM_020103 | - | - | **3.9** |
| B-cell leukemia/lymphoma 2 related protein A1 | *Bcl2a1* | NM_133416 | - | - | **3,9** |
| Fc receptor, IgG, low affinity III | *Fcgr3* | NM_053843 | - | - | **3,8** |
| immune-responsive gene 1 |  | XM_224476 | **-** | **-** | **4,5** |
| Lymphocyte antigen 6 complex, locus E | *Ly6E* | NM_001017467 | - | - | **4,2** |
| interleukin 1 beta | *Il1b* | NM_031512 | - | 1,8 | **4,1** |
| allograft inflammatory factor 1 | *Aif1* | NM_017196 | - | - | **3,7** |
| Ig gamma-1, chain C region |  | XM_234583 | - | - | **3,2** |
| immunoglobulin heavy chain 1a (serum IgG2a) | *Igh-1a* | XM_216800 | - | -1,6 | **3,1** |
| neutrophil cytosolic factor 1 | *Ncf1* | NM_053734 | - | - | **3,1** |
| myxovirus (influenza virus) resistance 2 | *Mx2* | NM_134350 | - | - | **3,1** |
| class III Fc-gamma receptor | *Fcgr3* | M64368 | - | - | **3,0** |
| immunoglobulin kappa light chain variable region |  | AF217588 | - | - | **3,0** |
| IgM kappa chain variable region {CDR1 to CDR3 region} | *S81289* |  | - | - | **2,9** |
| Ig variable region, light chain |  | XM_231999 | - | - | **2,9** |
| high affinity IgE receptor gamma chain |  | L04306 | - | - | **2,9** |
| CD68 antigen | *CD68* | XM_213372 | - | - | **2,8** |
| immunoglobulin joining chain | *Igj* | XM_341195 | - | - | **2,8** |
| immunoglobulin kappa-chain |  | XM_575546 | - | - | **2,8** |
| immunoglobulin rearranged kappa-chain mRNA variable ragion |  | L07408 | - | - | **2,8** |
| lymphocyte antigen 6 complex, locus E | *Ly6E* | NM_001017467 | - | - | **2,7** |
| clone 122.33 immunoglobulin kappa light chain variable region mRNA |  | AF217587 | - | - | **2,7** |
| R.norvegicus Ig rearranged light chain V-region mRNA, partial cds. |  | M61884 | - | - | **2,6** |
| clone 122.77 immunoglobulin kappa light chain variable region mRNA |  | AF217590 | - | - | **2,6** |
| gamma-2a immunoglobulin heavy chain | *IgG-2a* | NM_001014081 | - | - | **2,5** |
| Igh-6 protein |  | XM_216805 | - | -1,5 | **2,5** |
| R.norvegicus immunoglobulin kappa chain variable region. |  | X60291 | - | - | **2,5** |
| NGF-binding Ig rearranged L-chain mRNA, V-region, partial cds | *Igkv28* | L17078 | - | - | **2,5** |
| glucosaminyl (N-acetyl) transferase 3, mucin type | *Gcnt3* | NM_173312 | - | - | **2,5** |
| immunoglobulin rearranged kappa-chain mRNA variable |  | L07410 | - | - | **2,5** |
| ficolin B | *Fcnb* | NM_053634 | - | - | **2,4** |
| activated B cell RT1Bl alpha chain mRNA, complete cds. | *RT1-Ba* | AF307302 | - | - | **2,4** |
| Rat anti-neuropeptide substance P IgK chain NC1/34HL mRNA V-region. |  | M62828 | - | - | **2,4** |
| S100 calcium binding protein A8 | *S100a8* | NM_053822 | - | 1,8 | **2,4** |
| RT1 class Ia, locus A2 | *RT1-A2* | NM_001008829 | - | - | **2,4** |
| RT1 class II, locus Bb | *RT1-Bb* | NM_001004084 | - | - | **2,4** |
| granzyme C; natural killer cell protease 4 |  | XM_224226 | - | - | **2,3** |
| immunoglobulin heavy chain variable region |  | XM_345738 | - | - | **2,3** |
| Rat Ig active kappa-chain mRNA VJ-region from immunocytoma IR162 |  | M15402 | - | - | **2,3** |
| NATURAL KILLER CELL PROTEASE 1 PRECURSOR |  | XM_224224 | - | - | **2,3** |
| interleukin 1 receptor antagonist gene | *Il1rn* | BC070930 | - | - | **2,3** |
| toll-like receptor 2 | *Tlr2* | NM_198769 | - | 1,5 | **2,3** |
| Apolipoprotein L3 | *ApoL-III* | XM_235463 | - | - | **2,3** |
| NGF-binding Ig rearranged kappa-chain mRNA, V-region |  | L17080 | - | - | **2,3** |
| interferon gamma induced GTPase | *Igtp* | XM_220451 | - | - | **2,2** |
| DEAD/H (Asp-Glu-Ala-Asp/His) box polypeptide | *RIG-I* | XM_216380 | - | - | **2,2** |
| granzyme-like protein III. | *Gzmb* | X76996 | - | - | **2,2** |
| transmembrane receptor FcgammaRIII-X |  | NM_207603 | - | - | **2,2** |
| RT1 class II, locus Db1 | *RT1-Db1* | NM_001008884 | - | - | **2,2** |
| RT1 class II, locus Db1 | *RT1-Db1* | NM_001008884 | - | - | **2,2** |
| interleukin-3 receptor B subunit |  | AW141130 | - | - | **2,2** |
| Retinoic acid receptor responder protein 1 |  | XM_227232 | - | - | **2,2** |
| lipopolysaccharide binding protein | *Lbp* | NM_017208 | 1,9 | 1,8 | **2,2** |
| secretory leukocyte protease inhibitor |  | XM_215940 | - | - | **2,2** |
| RT1 class I, CE16 | *RT1-CE16* | NM_001008839 | - | - | **2,1** |
| MHC class II antigen |  | NP516919 | - | - | **2,1** |
| Fc receptor, IgG, low affinity IIb | *Fcgr2b* | NM_175756 | **-** | **-** | **2,1** |
| Tnfaip2 protein |  | XM_216786 | **-** | **-** | **2,1** |
| immunoglobulin light chain CDR1 |  | M87788 | **-** | **-** | **2,1** |
| lymphocyte antigen 86 | *Ly86* | XM_225636 | **-** | **-** | **2,1** |
| MHC class II antigen |  | NP516920 | **-** | **-** | **2,1** |
| MHC class Ib RT1.S3 | *RT1.S3* | AF029241 | - | - | **2,0** |
| IG HEAVY CHAIN V REGION VH558 A1/A4 |  | XM_345754 | - | - | **2,0** |
| secretory leukocyte protease inhibitor | *Slpi* | NM_053372 | **-** | **-** | **2,0** |
| MHC class II RT1-D beta1 chain haplotype k |  | AJ003232 | **-** | **-** | **2,0** |
| CD2 antigen | *Cd2* | NM_012830 | - | - | **2,0** |
| immunoglobulin kappa light chain variable region |  | AF217591 | **-** | **-** | **2,0** |
| nuclear factor, interleukin 3 regulated | *Nfil3* | NM_053727 | **-2,1** | -1,6 | - |
| **Interferon** |  |  |  |  |  |
| Interferon-induced guanylate-binding protein 1 |  | XM_221883 | **2,4** | 1,9 | **2,2** |
| interferon gamma inducible protein | *Ifi47* | NM_172019 | 1,7 | **2,7** | **7,3** |
| guanylate binding protein 2, interferon-inducible | *Gbp2* | NM_133624 | 1,6 | **2,4** | **3,1** |
| interferon-inducible GTPase |  | XM_225909 | - | **-** | **6,2** |
| interferon-inducible GTPase |  | XM_225907 | - | **-** | **5,9** |
| interferon-stimulated protein |  | XM_216605 | - | 1,6 | **4,1** |
| interferon inducible protein 1 | *Ifi1* | NM_001012007 | - | 1,8 | **3,7** |
| signal transducer and activator of transcription 1 | *Stat1* | NM_032612 | - | 1,7 | **3,6** |
| signal transducer and activator of transcription 1 | *Stat1* | NM_032612 | - | 1,5 | **2,4** |
| interferon-induced protein with tetratricopeptide repeats 2 | *Ifit2* | NM_001024753 | 1,5 | 1,7 | **3,4** |
| interferon induced transmembrane protein 3 | *Ifitm3* | XM_341957 | - | - | **2,9** |
| interferon, alpha-inducible protein 27-like | *Ifi27l* | NM_130743 | - | - | **2,9** |
| indoleamine 2,3-dioxygenase | *Indo* | NM_023973 | - | - | **2,8** |
| 2'-5' oligoadenylate synthetase 2-like | *Oasl2* | NM_001009682 | - | 1,5 | **2,7** |
| interferon-induced protein with tetratricopeptide repeats 1 | *Ifit1* | NM_020096 | - | - | **2,6** |
| interferon gamma inducing factor binding protein | *Il18bp* | NM_053374 | - | - | **2,6** |
| 2',5'-oligoadenylate synthetase 1, 40/46kDa | *Oas1* | NM_138913 | - | - | **2,6** |
| signal transducer and activator of transcription 1 | *Stat1* | NM_032612 | - | - | **2,6** |
| interferon regulatory factor 7 |  | XM_215121 | - | 1,5 | **2,6** |
| interferon induced transmembrane protein 2 like |  | XM_215117 | - | - | **2,4** |
| interferon-inducible protein 203 |  | AW141066 | - | -1,5 | **2,1** |
| 2'-5' oligoadenylate synthetase 1I | *Oas1i* | NM_001009680 | - | - | **2,1** |
| interferon stimulated gene factor 3 gamma |  | XM_224190 | - | - | **2,0** |
| alpha-interferon |  | XM_233145 | **-** | **2,0** | **-** |
| **Proteolysis** |  |  |  |  |  |
| ubiquitin D | *Ubd* | NM_053299 | 1,7 | **2,5** | **15,2** |
| proteosome (prosome, macropain) subunit, beta type 9 | *Psmb9* | NM_012708 | - | **2,0** | **3,7** |
| serine protease |  | XM_220222 | - | **2,0** | **2,6** |
| proteosome (prosome, macropain) subunit, beta type 8 | *Psmb8* | NM_080767 | - | - | **3,6** |
| potential ubiquitin ligase | *Herc6* | XM_342700 | - | 1,7 | **3,3** |
| proteasome (prosome, macropain) subunit, beta type 10 | *Psmb10* | XM_214687 | - | 1,5 | **2,1** |
| **Chemoatraction** |  |  |  |  |  |
| chemokine C-X-C motif ligand 9 | *Cxcl9* | NM_145672 | - | - | **5,4** |
| chemokine C-C motif ligand 19 predicted) | *Ccl19* | XM_342824 | - | - | **5,4** |
| chemokine C-C motif ligand 2 | *Ccl2* | NM_031530 | - | - | **4,1** |
| chemokine C-X-C motif ligand 10 | *Cxcl10* | NM_139089 | - | - | **3,6** |
| chemokine C-C motif ligand 7 | *Ccl7* | NM_001007612 | - | - | **2,7** |
| chemokine C-C motif ligand 7 | *Ccl7* | NM_001007612 | - | - | **2,5** |
| Small inducible cytokine B13 precursor CXCL13 |  | BC088260 | - | - | **2,1** |
| chemokine C-C motif ligand 11 | *Ccl11* | NM_019205 | - | **2,1** | 1,5 |
| chemokine C-X-C motif ligand 11 | *Cxcl11* | NM_182952 | - | - | **2,0** |
| **Cell adhesion** |  |  |  |  |  |
| lectin, galactoside-binding, soluble, 3 binding protein | *Lgals3bp* | NM_139096 | - | - | **2,6** |
| decorin | *Dcn* | NM_024129 | - | - | **2,5** |
| vascular cell adhesion molecule 1 | *Vcam1* | NM_012889 | - | - | **2,5** |
| integrin alpha L | *Itgal* | XM_219349 | - | - | **2,2** |
| gap junction membrane channel protein alpha 1 | *Gja1* | NM_012567 | - | - | **2,1** |
| vimentin | *Vim* | NM_031140 | - | - | **2,1** |
| type XV collagen |  | XM_216399 | - | - | **2,0** |
| Integrin beta-2 precursor | *Cd18* | XM_228072 | - | - | **2,0** |
| cysteine knot superfamily 1, BMP antagonist 1 | *Cktsf1b1* | NM_019282 | - | - | **2,0** |
| procollagen, type I, alpha 2 | *Col1a2* | NM_053356 | - | - | **2,0** |
| selectin, lymphocyte | *Sell* | NM_019177 | - | - | **2,0** |
| sialic acid binding Ig-like lectin 5 |  | XM_218639 | **2,0** | 1,8 | - |
| **Complement** |  |  |  |  |  |
| serine (or cysteine) proteinase inhibitor | *Serping1* | NM_199093 | **-** | **-** | **4,6** |
| complement component 1, s subcomponent | *C1s* | NM_138900 | **-** | **-** | **3,3** |
| complement component 2 | *C2* | NM_172222 | **-** | **-** | **3,2** |
| complement component 3 | *C3* | NM_016994 | **-** | **-** | **2,9** |
| Complement component 1, q subcomponent, alpha polypeptide |  | NM_001008515 | **-** | **-** | **2,7** |
| complement component 1, r subcomponent |  | XM_242644 | **-** | **-** | **2,5** |
| complement receptor 1/2-specific monoclonal antibody Ig |  | AF220555 | **-** | **-** | **2,5** |
| serine (or cysteine) proteinase inhibitor | *Serping1* | NM_199093 | **-** | **-** | **2,3** |
| Complement component 1, q subcomponent, alpha polypeptide |  | NM_001008515 | **-** | **-** | **2,3** |
| coagulation factor 3 | *F3* | NM_013057 | **-** | **-** | **2,0** |
| **Other or unknown function** |  |  |  |  |  |
| D site albumin promoter binding protein | *Dbp* | NM_012543 | **3,4** | 1,6 | **-** |
| adipocyte complement related protein of 30 kDa | *Acdc* | NM_144744 | **2,4** | **2,6** | **2,7** |
| hemoglobin alpha, adult chain 1 | *Hba-a1* | NM_013096 | **2,4** | **-** | 1,5 |
| hemoglobin beta chain complex | *Hbb* | NM_033234 | **2,4** | **-** | 1,6 |
| zero beta-1 globin |  | NM_198776 | **2,3** | **-** | **-** |
| stefin 2-like |  | XM_221409 | **2,2** | **-** | **-** |
| EST293986 |  | AW143690 | **2,2** | 1,6 | **-** |
| olfactory receptor gene Olr377 | *Olr377* | NM_001001276 | **2,2** | 1,7 | **-** |
| AA819079 UI-R-A0-ao-b-02-0-UI.s1 |  | AA819079 | **2,2** | 1,6 | **-** |
| olfactory receptor gene Olr804 | *Olr804* | NM_001000852 | **2,2** | 1,6 | **-** |
| FMS-like tyrosine kinase 1 | *Flt1* | NM_019306 | **2,2** | 1,6 | 1,5 |
| stefin A1 |  | XM_221416 | **2,1** | - | - |
| ADAMTS-6 precursor |  | XM_342184 | **2,1** | 1,6 | 1,6 |
| RIKEN cDNA 3110037K17 | *Dcir3* | XM_342752 | **2,1** | 1,6 | 1,5 |
| Hypothetical protein KIAA0196 |  | XM_238532 | **2,1** | 1,5 | - |
| Parkin |  | XM_345055 | **2,1** | 1,5 | - |
| hypothetical protein MGC35182 |  | XM_218843 | **2,1** | 1,7 | - |
| RIKEN cDNA 1700081O22 |  | XM_343674 | **2,1** | 1,7 | - |
| G patch domain containing 2 | *Gpatc2* | NM_001011909 | **2,1** | 1,8 | - |
| LOC363186 |  | XM_347110 | **2,1** | 1,5 | - |
| protein phosphatase 2 | *Ppp2r2c* | NM_057116 | **2,1** | 1,7 | 1,5 |
| Unknown |  | TC464290 | **2,1** | 1,6 | 1,8 |
| stefin A2 protein |  | XM_221411 | **2,1** | - | - |
| ets-related protein 71 - mouse |  | XM_341830 | **2,1** | 1,7 | 1,5 |
| MGC31683 protein |  | XM_345603 | **2,1** | 1,6 | - |
| putative pheromone receptor |  | XM_344197 | **2,1** | 1,6 | - |
| Unknown |  | AW527241 | **2,1** | 1,7 | - |
| gremlin 1 homolog, cysteine knot superfamily | *Grem1* | NM_019282 | **2,0** | 1,7 | - |
| gap junction membrane channel protein alpha 6 | *Gja6* | NM_019308 | **2,0** | 1,8 | - |
| Unknown |  | AA925959 | **2,0** | 1,6 | - |
| Mammary cancer associated protein RMT-1 | *Rmt1* | NM_145088 | **2,0** | 1,7 | 1,6 |
| ADAM28 isoform-1 |  | XM_214221 | **2,0** | 1,5 | - |
| hypothetical protein DKFZp434P0316 |  | XM_213533 | **2,0** | 1,7 | **-** |
| AW920717 EST352021 Rat gene index |  | AW920717 | **2,0** | 1,5 | **-** |
| Unknown |  | AI576488 | **2,0** | 1,6 | **-** |
| Q7TQ12 |  | TC462931 | **2,0** | 1,5 | **-** |
| Unknown |  | AA819014 | **2,0** | 1,5 | **-** |
| Unknown |  | TC511288 | **2,0** | 1,5 | **-** |
| RIKEN cDNA C330023F11 |  | XM_222995 | **2,0** | 1,7 | **-** |
| oligodendrocyte transcription factor 2 | *Olig2* | XM_221668 | **2,0** | 1,6 | **-** |
| tigger transposable element derived 3 homolog | *Tigd3* | XM_219522 | **2,0** | 1,6 | 1,5 |
| stefin A2 | *Stfa2* | NM_001004129 | **2,0** | - | - |
| membrane-associated protein 17 | *Map17* | NM_130401 | **2,0** | - | - |
| Nerve growth factor, beta polypeptide | *Ngfb* | XM_227525 | **2,0** | 1,6 | - |
| Unknown |  | ENSRNOT00000029004 | **2,0** | 1,7 | 1,5 |
| RIKEN cDNA 2410002O22 gene |  | XM_215476 | **2,0** | 1,5 | - |
| leucine rich repeat containing 4 protein precursor | *Lrrc4* | NM_001037336 | **2,0** | 1,6 | - |
| poly (ADP-ribose) polymerase family, member 10 |  | XM_216963 | **2,0** | 1,6 | - |
| Unknown |  | TC495766 | **2,0** | 1,6 | 1,5 |
| phospholipase A2, group IB | *Pla2g1b* | NM_031585 | **2,0** | 1,5 | - |
| Epithelial protein lost in neoplasm | *eplin* | XM_217039 | **2,0** | 1,6 | - |
| Unknown |  | CB547383 | **2,0** | 1,6 | - |
| period homolog 2 | *Per2* | NM_031678 | **2,0** | - | - |
| persephin | *Pspn* | NM_013014 | **2,0** | 1,7 | - |
| ankyrin repeat and SOCs box-containing protein 5 |  | XM_341469 | **2,0** | 1,5 | - |
| Unknown |  | BI282062 | **2,0** | 1,5 | - |
| putative pheromone receptor |  | XM_218017 | **2,0** | 1,5 | - |
| nuclear receptor subfamily 1, group D, member 1 | *Nr1d1* | NM_145775 | **2,0** | - | - |
| inner nuclear membrane protein |  | XM_216900 | **2,0** | 1,6 | - |
| m-ephrin-B3 |  | XM_340824 | **2,0** | 1,7 | 1,5 |
| Unknown |  | CB544812 | **2,0** | 1,6 | - |
| Unknown |  | ENSRNOT00000037446 | **2,0** | 1,6 | - |
| Unknown |  | AA957618 | **2,0** | 1,6 | - |
| Eso3 protein |  | XM_215227 | **2,0** | 1,6 | - |
| Unknown |  | BF393108 | **2,0** | 1,7 | - |
| rhodopsin kinase | *Rhok* | NM_031096 | **2,0** | 1,6 | - |
| Unknown |  | BF555121 | **-** | **3,9** | - |
| Unknown |  | BM389675 | 1,8 | **3,3** | 1,6 |
| Unknown |  | AI234967 | 1,5 | **3,2** | **4,2** |
| Unknown |  | BF403483 | 1,8 | **3,1** | 1,5 |
| Unknown |  | BF543790 | 1,7 | **3,0** | 1,5 |
| hypothetical protein FLJ37300 |  | XM_221082 | 1,7 | **3,0** | 1,5 |
| schlafen 3 | *Slfn3* | NM_053687 | - | **2,7** | **6,8** |
| alpha-2,3-sialyltransferase ST3Gal IV | *siat4c* | NM_203337 | 1,6 | **2,7** | 1,8 |
| Unknown |  | TC489033 | 1,7 | **2,7** | **-** |
| receptor-interacting serine-threonine kinase 3 | *Ripk3* | NM_139342 | 1,5 | **2,7** | **4,6** |
| Unknown |  | A_44_P985331 | 1,5 | **2,5** | **-** |
| delta-like 1 homolog | *Dlk1* | NM_053744 | 1,6 | **2,4** | **-** |
| hypothetical protein MGC32065 |  | XM_236271 | 1,5 | **2,4** | 1,7 |
| Unknown |  | AI059874 | 1,5 | **2,3** | **-** |
| EST227090 |  | AI230395 | 1,5 | **2,3** | **-** |
| Unknown |  | TC486825 | - | **2,3** | **-** |
| protein phosphatase 1, regulatory (inhibitor) subunit 14c | *Ppp1r14c* | NM_133425 | - | **2,3** | **-** |
| RNA-binding region containing 1 | *RNP1* | XM_345477 | - | **2,3** | **-** |
| LNX2 |  | XM_342997 | 1,5 | **2,3** | **-** |
| Unknown |  | BF564613 | 1,5 | **2,2** | **-** |
| Tripartite motif protein 15 (Zinc finger protein B7) |  | XM_227945 | **-** | **2,1** | **3,0** |
| nucleotide-binding oligomerization domains 27 |  | XM_344723 | **-** | **2,1** | **-** |
| 5-hydroxytryptamine (serotonin) receptor 5A | *Htr5a* | NM_013148 | **-** | **2,1** | **-** |
| pleiomorphic adenoma gene-like 1 | *Plagl1* | NM_012760 | **-** | **2,1** | **-** |
| olfactory receptor gene Olr233 | *Olr233* | NM_001000207 | **-** | **2,0** | **-** |
| olfactory receptor gene Olr232 | *Olr232* | NM_001001035 | **-** | **2,0** | **-** |
| MAS-related G protein-coupled receptor, member B2 | *Mrgprb2* | NM_001002285 | **-** | **2,0** | **-** |
| chitinase 3-like 1 (cartilage glycoprotein-39) | *Chi3l1* | XM_341123 | **-** | **-** | **3,9** |
| OEF2 | *OEF2* | XM_575365 | - | 1,6 | **3,9** |
| CDNA sequence BC023105 |  | XM_225905 | - | 1,7 | **3,8** |
| Unknown |  | CO565948 | - | 1,9 | **3,7** |
| glycoprotein (transmembrane) | *Gpnmb* | NM_133298 | - | - | **3,7** |
| Unknown |  | BF542741 | - | - | **3,6** |
| arginosuccinate synthetase | *Ass* | NM_013157 | - | - | **3,5** |
| NADPH oxidase beta subunit gp91phox |  | TC551872 | - | - | **3,4** |
| Replication enhancer protein |  | TC555318 | - | 1,8 | **3,4** |
| mRNA for monoclonal antibody Y13-259 Vk. | *X55180* | X55180 | - | - | **3,3** |
| matrix Gla protein | *Mgp* | NM_012862 | - | - | **3,3** |
| putative ISG12 (b) protein | *isg12(b)* | XM_238467 | 1,5 | 1,7 | **3,3** |
| gamma-glutamyltransferase 1 | *Ggt1* | NM_053840 | 1,7 | 1,9 | **3,2** |
| Rat libraries cDNA clone Contig294 |  | CF110082 | - | - | **3,1** |
| Unknown |  | ENSRNOT00000036096 | - | - | **3,1** |
| AW917984 EST349288 Rat gene index |  | AW917984 | - | - | **2,9** |
| hydroxysteroid 11-beta dehydrogenase 1 | *Hsd11b1* | NM_017080 | - | - | **2,9** |
| SPI6 |  | NM_001007732 | - | - | **2,8** |
| Apolipoprotein L3 |  | XM_235461 | - | - | **2,8** |
| membrane-spanning 4-domains, subfamily A, member 11 | *Ms4a11* | XM_342028 | - | - | **2,8** |
| Unknown |  | ENSRNOT00000030371 | - | - | **2,7** |
| membrane-spanning 4-domains, subfamily A, member 6B | *Ms4a6b* | NM_001006975 | - | - | **2,7** |
| hypothetical protein MGC29390 |  | XM_221401 | - | 1,7 | **2,7** |
| Unknown |  | CB547712 | - | - | **2,7** |
| EST349927 |  | AW918623 | - | - | **2,5** |
| hydroxysteroid 11-beta dehydrogenase 1 | *Hsd11b1* | NM_017080 | - | - | **2,5** |
| lumican | *Lum* | NM_031050 | - | - | **2,4** |
| hypothetical protein MGC29390 |  | XM_221401 | - | 1,5 | **2,4** |
| Tyro protein tyrosine kinase binding protein | *Tyrobp* | NM_212525 | - | - | **2,3** |
| Unknown |  | ENSRNOT00000032063 | - | - | **2,3** |
| Unknown |  | TC515376 | - | - | **2,3** |
| schlafen1 |  | XM_343949 | - | - | **2,3** |
| UI-R-E0-cv-d-05-0-UI.s1 |  | AI502476 | - | - | **2,3** |
| GTPase, IMAP family member 4 | *Gimap4* | NM_173153 | - | - | **2,3** |
| hexokinase 3 | *Hk3* | NM_022179 | - | - | **2,3** |
| Unknown |  | TC485544 | - | - | **2,3** |
| Unknown |  | ENSRNOT00000032931 | - | - | **2,3** |
| mac25 |  | XM_214014 | - | - | **2,3** |
| Unknown |  | ENSRNOT00000028037 | - | - | **2,3** |
| EST453951 |  | BF289360 | - | - | **2,3** |
| Carbonic anhydrase 3 | *Ca3* | NM_019292 | 1,9 | 1.5 | **2,2** |
| pleckstrin |  | XM_344267 | - | - | **2,2** |
| UDP-Gal:betaGlcNAc beta 1,4- galactosyltransferase, polypeptide 1 | *B4galt1* | XM_342820 | - | - | **2,2** |
| protein tyrosine phosphatase, receptor type, C | *Ptprc* | NM_138507 | - | -1,6 | **2,2** |
| pleckstrin homology-like domain, family A, member 1 | *Phlda1* | NM_017180 | - | 1,6 | **2,2** |
| cathepsin Y |  | NM_183330 | - | - | **2,2** |
| Unknown |  | CB545779 | - | - | **2,2** |
| SRrp35 |  | XM_216364 | - | - | **2,2** |
| insulin-like growth factor binding protein 4 | *Igfbp4* | NM_001004274 | - | - | **2,2** |
| NGF-binding Ig light chain |  | XM_575534 | - | - | **2,2** |
| EST293671 |  | AW143375 | - | - | **2,2** |
| protein tyrosine phosphatase, receptor type, C | *Ptprc* | Y00065 | - | - | **2,2** |
| membrane-spanning 4-domains, subfamily A, member 6A | *Ms4a6a* | XM_215145 | - | - | **2,2** |
| secreted acidic cysteine rich glycoprotein | *Sparc* | NM_012656 | 1,6 | - | **2,2** |
| lymphocyte cytosolic protein 1 |  | XM_224406 | - | - | **2,1** |
| deoxyribonuclease I-like 3 | *Dnase1l3* | NM_053907 | - | -1,5 | **2,1** |
| stearoyl-Coenzyme A desaturase 1 | *Scd1* | NM_139192 | - | 1,6 | **2,1** |
| mitogen activated protein kinase 13 | *Mapk13* | NM_019231 | 1,5 | 1,7 | **2,1** |
| cystatin A | *Csta* | XM_213616 | - | 1,5 | **2,1** |
| olfactomedin 4 | *Olfm4* | XM_224413 | - | - | **2,1** |
| Serine protease inhibitor | *Spin2c* | NM_031531 | 1,6 | - | **2,1** |
| RIKEN cDNA 9030625A04 |  | XM_233749 | - | - | **2,1** |
| EST345547 |  | AW914230 | - | - | **2,1** |
| Fatty acid-binding protein, epidermal |  | XM_231258 | - | - | **2,0** |
| Fatty acid binding protein 5, epidermal | *Fabp5* | NM_145878 | - | 1,5 | **2,0** |
| Fatty acid binding protein 4, adipocyte | *Fabp4* | NM_053365 | 1,7 | 1,7 | **2,0** |
| 5830458K16Rik protein |  | XM_341005 | - | - | **2,0** |
| caspase 12 | *Casp12* | NM_130422 | - | 1,5 | **2,0** |
| Unknown |  | TC488814 | - | - | **2,0** |
| membrane-spanning 4-domains, subfamily A, member 11 | *Ms4a11* | XM_342028 | - | - | **2,0** |
| Unknown |  | CA504564 | - | - | **2,0** |
| pleckstrin homology-like domain, family A, member 2 | *Phlda2_predicted* | XM_215132 | - | - | **2,0** |
| RIKEN cDNA 1600029D21 |  | XM_343387 | - | - | **2,0** |
| Unknown |  | BQ207049 | - | - | **2,0** |
| Beta-arrestin 2 | *Arrb2-ps* | XM_345084 | - | - | **2,0** |
| Unknown |  | TC479507 | - | - | **2,0** |
| platelet derived growth factor receptor, alpha polypeptide | *Pdgfra* | XM_214030 | - | - | **2,0** |
| Unknown |  | TC500789 | - | - | **2,0** |
| Unknown |  | ENSRNOT00000004831 | - | 1,5 | **2,0** |
| Unknown |  | TC464953 | - | - | **2,0** |
| Unknown |  | TC510047 | - | 1,5 | **2,0** |
| EST451900 |  | BF287204 | - | - | **2,0** |
| B aggressive lymphoma |  | XM_221404 | - | - | **2,0** |
| ceruloplasmin | *Cp* | NM_012532 | - | - | **2,0** |
| onzin |  | XM_341188 | 1,5 | - | **2,0** |
| Unknown |  | BF567631 | - | - | **2,0** |
| Unknown |  | TC489287 | - | - | **2,0** |
| RIKEN cDNA 4933417L10 |  | XM_344412 | - | - | **2,0** |
| Unknown |  | ENSRNOT00000029142 | - | - | **2,0** |
| Unknown |  | BE109691 | - | - | **2,0** |
| Unknown |  | BC088253 | - | - | **2,0** |
| Fatty acid binding protein 2, intestinal | *Fabp2* | NM_013068 | -1,6 | -1,5 | **-2,0** |
| Unknown |  | BF290649 | - | -1,6 | **-2,0** |
| Unknown |  | BE109736 | - | - | **-2,0** |
| metallothionein-2 and metallothionein-1 |  | M11794 | - | -1,5 | **-2,1** |
| Mss4 protein | *Mss4* | NM_001007678 | - | -1,6 | **-2,1** |
| retinoid binding protein 7 |  | XM_575960 | -1,7 | - | **-2,1** |
| Fatty acid binding protein 1, liver | *Fabp1* | NM_012556 | **-2,3** | **-2,3** | -1,5 |
| aldehyde dehydrogenase family 1, member A1 | *Aldh1a1* | NM_022407 | **-** | **-2,0** | **-2,8** |
| Unknown |  | ENSRNOT00000024093 | **-** | **-2,2** | **-2,4** |
| resistin-like gamma | *Retnlg* | NM_181625 | **-** | **-3,2** | **-2,2** |
| resistin like alpha | *Retnla* | NM_053333 | **-** | **-3,2** | **-2,1** |
| regenerating islet-derived family, member 4 | *Reg4* | NM_001004096 | **-2,0** | **-2,6** | **-** |
| membrane protein, palmitoylated 2 | *Mpp2* | XM_340912 | **-2,1** | -1,8 | -1,8 |
| Unknown | *AI029975* | AI029975 | **-2,2** | - | - |
| aryl hydrocarbon receptor nuclear translocator-like | *Arntl* | NM_024362 | **-2,6** | -1,7 | - |
| ADP-ribosylation factor guanine nucleotide-exchange factor 6 | *Sec7* | XM_231031 | **-2,7** | **-3,0** | **-2,7** |
