# Supplementary material for: Salmonella induces prominent gene expression in the rat colon
Source: BMC Microbiol. 2007 Sep 12;7:84. doi: 10.1186/1471-2180-7-84 (PMC2048963; doi:10.1186/1471-2180-7-84)
Supplement: Additional file 2 — Housekeeping and cell type specific genes. The fold change in expression of housekeeping genes and cell-type specific genes in the colon mucosa at days 1, 3 and 6 after oral Salmonella infection. [file 1471-2180-7-84-S2.doc]

Additional file 2

The fold change in expression of housekeeping genes and cell-type specific genes in the colon mucosa at days 1, 3 and 6 after oral *Salmonella* infection.

| Gene Name | | Gene symbol | Sequence ID | Fold Change infected vs non-infected rats on different days p.i. | | | |
| --- | --- | --- | --- | --- | --- | --- | --- |
|  |  |  |  | Time course infection study | | | Dietary infection study^a^ |
|  | |  |  | Day 1 | Day 3 | Day 6 | Day 2 |
| **Housekeeping genes** | |  |  |  |  |  |  |
| Actin beta | | Actb | NM_031144 | 1.1 | 1.2 | 1.1 | 1.2 |
| Glyceraldehyde-3-phosphate dehydrogenase | | Gapdh | NM_017008 | 1.0 | 1.0 | 1.0 | 1.0 |
| Aldolase a, fructose-biphosphate | | Aldoa | NM_01495 | 1.1 | 1.0 | 1.0 | -1.1 |
| Phosphoglucerate kinase 1 | | Pgk | NM_053291 | 1.0 | 1.2 | 1.2 | 1.1 |
| Adp-ribosylation factor 1 | | Arf | NM_022518 | 1.0 | 1.0 | 1.0 | 1.1 |
| H3 histone, family 3B | | H3f3b | NM_053985 | 1.0 | -1.1 | 1.0 | 1.0 |
| Succinate dehydrogenase complex, subunit A | | Sdha | NM_0130428 | 1.0 | -1.1 | -1.1 | 1.0 |
| Ribosomal protein S26 | | Rps26 | NM_013224 | -1.1 | -1.1 | 1.0 | 1.1 |
| Pleckstrin homology domain-containing family A member 3 | | Plekha3 | NM_001013077 | 1.0 | -1.1 | 1.0 | 1.1 |
| **Cell type specific genes** | |  |  |  |  |  |  |
| *Enterocyte* | |  |  |  |  |  |  |
| Intestinal fatty acid binding protein 2 | | Fabp2 | NM_013068 | -1.6 | -1.5 | -2.0 | -1.5 |
| Villin 2 | | Vil2 | NM_019357 | 1.2 | 1.1 | 1.0 | 1.0 |
| Intestinal alkaline phosphatase-II gene | | Alpi2 | NM_022680 | 1.1 | 1.0 | -1.0 | -1.3 |
| *Goblet Cell* | |  |  |  |  |  |  |
| Colonic mucin 2 | | Muc2 | TC556623 | -1.1 | -1.0 | -1.1 | -1.1 |
| Mucin 3 | | Muc3 | U76551 | 1.7 | 1.3 | 1.3 | 1.0 |
| Trefoil factor 1 | | Tff1 | NM_057129 | -1.0 | -1.0 | -1.2 | -1.2 |
| Trefoil factor 3 | | Tff3 | NM_013042 | 1.1 | -1.4 | 1.0 | 1.2 |
| Chloride channel calcium activated 3 | | Clca3 | XM_217689 | 1.1 | -1.5 | -1.2 | -1.2 |
| Chloride channel calcium activated 6 | | Clca6 | NM_201419 | 2.3 | 2.2 | 3.7 | 2.3 |
| Sialyltransferase 8 B | | Siat8b | NM_057156 | -1.0 | 1.0 | 1.0 | ND |
| Sialyltransferase 9 | | Siat9 | NM_031337 | 1.1 | 1.0 | 1.0 | 1.2 |
| *Leukocytes* |  | |  |  |  |  |  |
| Leukocyte cell derived chemotaxin 1 | Lect1 | | NM_030854 | 1.2 | 1.2 | 1.1 | 1.1 |
| CD84 leukocyte antigen | CD84 | | XM_577290 | 1.1 | -1.0 | 1.5 | ND |
| SP140 nuclear body protein | Sp140 | | XM_237361 | 1.1 | -1.0 | 1.2 | 1.1 |
| Leucocyte specific transcript 1 | Lst1 | | NM_022634 | -1.0 | 1.2 | 1.1 | -1.0 |
| RT1 class I, CE12 | RT1-CE12 | | XM_227986 | 1.2 | 1.0 | 1.5 | 1.1 |
| RT1 class Ib, locus Aw2 | RT1-Aw2 | | Y13890 | 1.1 | -1.0 | 1.6 | 1.0 |
| Granzyme A | Gzma | | NM_153468 | 1.0 | -1.0 | 1.7 | 1.0 |
| Granzyme B | Gzmb | | NM_138517 | -1.0 | -1.0 | 1.1 | 1.1 |
| Granzyme C | Gzmc | | NM_134332 | 1.1 | 1.0 | 1.3 | -1.1 |

^a^ Fold Change infected vs non-infected rats fed a cellulose diet at day 2 p.i (obtained from the dietary infection study). Genes not detected in this independent study are indicated by ND.
