# Supplementary material for: Salmonella induces prominent gene expression in the rat colon
Source: BMC Microbiol. 2007 Sep 12;7:84. doi: 10.1186/1471-2180-7-84 (PMC2048963; doi:10.1186/1471-2180-7-84)
Supplement: Additional file 3 — Dietary modulated genes. Processes regulated in colon by Salmonella at day 2 in cellulose fed and FOS fed rats. [file 1471-2180-7-84-S3.doc]

Additional file 3

Processes regulated in colon by *Salmonella* at day 2 in cellulose fed and FOS fed rats.

|  | Gene Name | Gene symbol | Sequence ID | Fold Change infected vs non-infected rats^a^ | |
| --- | --- | --- | --- | --- | --- |
|  |  |  |  | Cellulose diet | FOS diet |
| **Overlap (17 genes)** | |  |  |  |  |
|  | ***Transport*** |  |  |  |  |
|  | Chloride channel calcium activated 6 | *Clca6* | NM_201419 | **2.3** | **3.9** |
|  | ***Oxidative stress*** |  |  |  |  |
|  | Glutathione peroxidase 2 | *Gpx2* | NM_183403 | **2.2** | **4.2** |
|  | ***Immune response, Antimicrobial defense and Inflammatory response*** |  |  |  |  |
|  | Pancreatitis-associated protein | *Pap* | NM_053289 | **17.7** | **35.6** |
|  | Phospholipase A2, group IIA (platelets, synovial fluid) | *Pla2g2a* | NM_031598 | **7.3** | **18.9** |
|  | Interleukin 1 beta | *Il1b* | NM_031512 | **2.6** | **3.5** |
|  | TRAF2 binding protein | *Traf2bp* | NM_001014044 | **2.4** | **3.4** |
|  | Chemokine (C-C motif) ligand 2 | *Ccl2* | NM_031530 | **2.8** | **3.2** |
|  | Chemokine (C-X-C motif) ligand 10 | *Cxcl10* | NM_139089 | **2.3** | **3.2** |
|  | ***Interferon*** |  |  |  |  |
|  | Interferon gamma inducible protein | *Ifi47* | NM_172019 | **2.6** | **5.2** |
|  | Interferon-inducible GTPase | *Iigp1* | NM_001012353 | **2.4** | **3.4** |
|  | ***Proteolysis*** |  |  |  |  |
|  | Ubiquitin D | *Ubd* | NM_053299 | **3.4** | **7.6** |
|  | Proteosome (prosome, macropain) subunit, beta type 9 | *Psmb9* | NM_012708 | **2.0** | **3.1** |
|  | ***Miscellaneous*** |  |  |  |  |
|  | Unknown | *-* | BF555121 | **3.9** | **10.4** |
|  | Unknown | *-* | TC555318 | **2.4** | **4.2** |
|  | Unknown | *-* | AI234967 | **2.7** | **3.9** |
|  | Palmitoyl-protein thioesterase | *Ppt* | XM_342904 | **2.8** | **3.9** |
|  | Neurexin 2 | *Nrxn2* | NM_053846 | **-3.3** | **-3.1** |
| **Cellulose only (3 genes)** | |  |  |  |  |
|  | Protease, serine, 3 (mesotrypsin) | *Prss3* | XM_342668 | **10.7** | -1.7 |
|  | Unknown | *-* | TC526384 | **2.2** | 1.5 |
|  | Unknown | *-* | NM_001014241 | **-4.4** | -1.4 |
| **FOS only (58 genes)** | |  |  |  |  |
|  | ***Transport*** |  |  |  |  |
|  | Solute carrier family 10, member 2 | *Slc10a2* | NM_017222 | 1.2 | **2.1** |
|  | ***Oxidative stress*** |  |  |  |  |
|  | Dual oxidase 2 | *Duox2* | NM_024141 | 1.6 | **2.9** |
|  | Xanthine dehydrogenase | *Xdh* | NM_017154 | 1.4 | **2.7** |
|  | ***Immune response, Antimicrobial defense and Inflammatory response*** |  |  |  |  |
|  | Myxovirus (influenza virus) resistance 2 | *Mx2* | NM_134350 | 1.4 | **3.0** |
|  | Schlafen 3 | *Slfn3* | NM_053687 | 2.0 | **3.0** |
|  | Interleukin 1 beta | *Il1b* | NM_031512 | 1.9 | **2.6** |
|  | Serum amyloid A 3 | *Saa3* | AY325259 | 1.4 | **2.5** |
|  | Regenerating islet-derived 3 gamma | *Reg3g* | NM_173097 | 1.9 | **2.4** |
|  | Fatty acid binding protein 5, epidermal | *Fabp5* | NM_145878 | 1.6 | **2.3** |
|  | Lymphocyte antigen 6 complex, locus E | *Ly6e* | NM_001017467 | 1.5 | **2.3** |
|  | Chemokine (C-C motif) ligand 7 | *Ccl7* | NM_001007612 | 2.0 | **2.2** |
|  | Lipopolysaccharide binding protein | *Lbp* | NM_017208 | 1.3 | **2.0** |
|  | Interleukin 1 alpha | *Il1a* | NM_017019 | 1.4 | **2.0** |
|  | Toll-like receptor 2 | *Tlr2* | NM_198769 | 1.3 | **2.0** |
|  | ***Interferon*** |  |  |  |  |
|  | Interferon-inducible GTPase | *Iigp* | NM_001024884 | 1.7 | **3.7** |
|  | guanylate nucleotide binding protein 2 | *Gbp2* | NM_133624 | 1.7 | **3.4** |
|  | signal transducer and activator of transcription 1 | *Stat1* | NM_032612 | 1.8 | **3.0** |
|  | interferon, alpha-inducible protein | *G1p2* | XM_216605 | 1.5 | **2.6** |
|  | indoleamine 2,3-dioxygenase | *Indo* | NM_023973 | 1.5 | **2.3** |
|  | interferon gamma induced GTPase | *Igtp* | XM_220451 | 1.3 | **2.3** |
|  | interferon inducible protein 1 | *Ifi1* | NM_001012007 | 1.4 | **2.3** |
|  | Interferon regulatory factor 7 | *Irf7* | NM_001033691 | 1.3 | **2.2** |
|  | EF hand domain containing 2 | *Efhd2* | NM_001031648 | 1.3 | **2.1** |
|  | ***Proteolysis*** |  |  |  |  |
|  | transglutaminase 2, C polypeptide | *Tgm2* | NM_019386 | 1.8 | **4.4** |
|  | proteosome (prosome, macropain) subunit, beta type 8 | *Psmb8* | NM_080767 | 1.7 | **2.7** |
|  | protease, serine, 22 | *Prss22* | XM_220222 | 1.6 | **2.4** |
|  | serine (or cysteine) peptidase inhibitor, clade G, member 1 | *Serping1* | NM_199093 | 1.7 | **2.4** |
|  | potential ubiquitin ligase | *Herc6* | XM_342700 | 1.5 | **2.4** |
|  | carboxypeptidase B gene, exons 6, 7, and 8 | *Carb7* | M23953 | 1.5 | **2.2** |
|  | stefin A2 | *Stfa2* | NM_001004129 | 1.2 | **2.2** |
|  | parkin | *Park2* | NM_020093 | 1.4 | **2.1** |
|  | legumain | *Lgmn* | NM_022226 | 1.4 | **2.1** |
|  | stefin 2-like | *Stf2* | XM_221409 | 1.2 | **2.1** |
|  | proteasome (prosome, macropain) subunit, beta type 10 | *Psmb10* | NM_001025637 | 1.5 | **2.0** |
|  | ***Miscellaneous*** |  |  |  |  |
|  | Unknown | *-* | XM_225905 | 1.6 | **3.6** |
|  | adipocyte complement related protein/ Adiponectin | *Acdc* | NM_144744 | 1.3 | **2.9** |
|  | Unknown | *-* | XM_221401 | 1.5 | **2.6** |
|  | tripartite motif protein 15 | *Trim15* | XM_227945 | 1.6 | **2.4** |
|  | matrix Gla protein | *Mgp* | NM_012862 | 1.7 | **2.4** |
|  | glucosaminyl (N-acetyl) transferase 3, mucin type | *Gcnt3* | NM_173312 | 1.8 | **2.4** |
|  | membrane-spanning 4-domains, subfamily A, member 12 | *Ms4a12* | XM_219588 | 1.4 | **2.4** |
|  | receptor-interacting serine-threonine kinase 3 | *Ripk3* | NM_139342 | 1.4 | **2.4** |
|  | Unknown | *-* | XM_221376 | 1.4 | **2.3** |
|  | Unknown |  | CO402999 | 1.4 | **2.3** |
|  | zymogen granule protein 16 | *Zg16* | NM_134409 | 1.2 | **2.2** |
|  | histidyl tRNA synthetase 2 | *Hars2* | BQ207049 | 1.2 | **2.2** |
|  | Unknown | *-* | AA924770 | 1.1 | **2.1** |
|  | round spermatids protein STDP2 | *Stdp2* | XM_573991 | 1.2 | **2.1** |
|  | Unknown | *-* | AW920888 | 1.3 | **2.1** |
|  | B-cell leukemia/lymphoma 2 related protein A1 | *Bcl2a1* | NM_133416 | 1.7 | **2.1** |
|  | GLI pathogenesis-related 2 | *Glipr2* | XM_342827 | 1.4 | **2.1** |
|  | Unknown | *-* | XM_223906 | 1.2 | **2.1** |
|  | Unknown | *-* | XM_575189 | 1.4 | **2.1** |
|  | Unknown | *-* | NM_001014209 | 1.5 | **2.0** |
|  | MAp19 protein | *-* | Y18568 | 1.2 | **2.0** |
|  | basic transcription element binding protein 1 | *Bteb1* | NM_057211 | 1.3 | **2.0** |
|  | Unknown | *-* | BF281337 | 1.2 | **2.0** |
|  | period homolog 2 (Drosophila) | *Per2* | NM_031678 | -1.1 | **-2.2** |

^a^ Values in bold exceed cut-off value FC>2 or FC<-2.
