# Supplementary material for: Salmonella induces prominent gene expression in the rat colon
Source: BMC Microbiol. 2007 Sep 12;7:84. doi: 10.1186/1471-2180-7-84 (PMC2048963; doi:10.1186/1471-2180-7-84)
Supplement: Additional file 5 — Primer sequences. Sequences of the primers used for Q-PCR analysis. [file 1471-2180-7-84-S5.doc]

Additional file 5

Sequences of the primers used for Q-PCR analysis

| **Gene symbol** | **Sequence ID** | **Forward primer (5' → 3')** | **Reverse primer (5' → 3')** | **product length** |
| --- | --- | --- | --- | --- |
| *Actin β* | NM_031144 | CTTTCTACAATGAGCTGCGTGTG | GTCAGGATCTTCATGAGGTAGTCTGTC | 315 |
| *Arf1* | NM_022518 | CTCCGAGATGCCGTTCTCTTG | GGTAGCCTGAATGTACCAGTTCC | 128 |
| *Clca6* | NM_201419 | GATGGGGAAAAGATCAGTCTAACATGG | GTTTCTGGTTTAAAGGCAAAGGTTTCC | 200 |
| *Gbp2* | XM_225909 | GACCTCAAGCCTAGAGCACAC | GACTTCAAGCAAATAAAGCCACAG | 106 |
| *Gpx2* | NM_183403 | GCCTAGTGGTTCTCGGCTTCC | AGGGTAGGGCAGCTTGTCTTTC | 200 |
| *Ifi47* | NM_172019 | GTGCGGTTGGTGGTGGTTG | CCGAGTCTGTTGCTCACTGC | 83 |
| *Il1b* | NM_031512 | AAGGGGTTGAATCTATACCTGTCCTG | TGCTCTGCTTGAGAGGTGCTG | 200 |
| *Pap* | NM_053289 | CTGCCAGAAGAGACCTGAAGGAC | CACCTCCATTGGGTTCTCCACC | 154 |
| *Pla2g2a* | NM_031598 | CAAAGTTTCTGACCTACAAGTTCTCCTAC | CTTTCAGCAACTGGGCGTCTTC | 200 |
| *Rps29* | NM_012876 | CCGACAGTGCTTCCGTCAG | GACAGTTGGTTTCATTGGGTAGAC | 102 |
| *Stat1* | NM_032612 | GTTCGCCACCATCCGCTTC | TCTTCCTCTCCTCCTTCAGACAG | 200 |
| *Tgm2* | NM_019386 | CACTTTCTGATTCCCTGTATGACTGTG | ACCCTTGACCGACTTCAGCTTG | 200 |
